# Large-scale Proteomics Combined with Transgenic Experiments Demonstrates An Important Role of Jasmonic Acid in Potassium Deficiency Response in Wheat and Rice*[Fn FN2]

**DOI:** 10.1074/mcp.RA117.000032

**Published:** 2017-08-18

**Authors:** Gezi Li, Yufang Wu, Guoyu Liu, Xianghong Xiao, Pengfei Wang, Tian Gao, Mengjun Xu, Qiaoxia Han, Yonghua Wang, Tiancai Guo, Guozhang Kang

**Affiliations:** From the ‡National Key Laboratory of Wheat and Maize Crop Science, Zhengzhou, 450002, China;; §Collaborative Innovation Center of Henan Food Crops, Henan Agricultural University, Zhengzhou, 450002, China;; ¶National Engineering Research Centre for Wheat, Henan Agricultural University, Zhengzhou, 450002, China

## Abstract

Potassium (K^+^) is the most abundant inorganic cation in plants, and molecular dissection of K^+^ deficiency has received considerable interest in order to minimize K^+^ fertilizer input and develop high quality K^+^-efficient crops. However, the molecular mechanism of plant responses to K^+^ deficiency is still poorly understood. In this study, 2-week-old bread wheat seedlings grown hydroponically in Hoagland solution were transferred to K^+^-free conditions for 8 d, and their root and leaf proteome profiles were assessed using the iTRAQ proteome method. Over 4000 unique proteins were identified, and 818 K^+^-responsive protein species showed significant differences in abundance. The differentially expressed protein species were associated with diverse functions and exhibited organ-specific differences. Most of the differentially expressed protein species related to hormone synthesis were involved in jasmonic acid (JA) synthesis and the upregulated abundance of JA synthesis-related enzymes could result in the increased JA concentrations. Abundance of allene oxide synthase (AOS), one key JA synthesis-related enzyme, was significantly increased in K^+^-deficient wheat seedlings, and its overexpression markedly increased concentrations of K^+^ and JA, altered the transcription levels of some genes encoding K^+^-responsive protein species, as well as enhanced the tolerance of rice plants to low K^+^ or K^+^ deficiency. Moreover, rice AOS mutant (*osaos*) exhibited more sensitivity to low K^+^ or K^+^ deficiency. Our findings could highlight the importance of JA in K^+^ deficiency, and imply a network of molecular processes underlying plant responses to K^+^ deficiency.

Potassium (K^+^) is the most abundant inorganic cation in plants, comprising up to 10% of a plant's dry weight (1). It plays a major role in several physiological processes, including photosynthesis, osmoregulation, enzyme activation, protein synthesis, ion homeostasis, and maintenance of the anion-cation balance (2, 3). K^+^ represents the fourth most abundant element in the lithosphere; however, only a low proportion (1–4%) on the surface of clay humus particles in soil is bioavailable (4). The major fraction is part of mineral compositions and is unavailable to plants; thus, millions of tons of potassium fertilizers must be applied to fields annually worldwide to increase crop productivity (5). However, more than 50% of applied chemical fertilizers, including potassium, is wasted because of the low mineral nutrient efficiency of crops (5, 6). Therefore, it is essential to minimize K^+^ fertilizer input and develop high quality K^+^-efficient crops, which require a detailed understanding on the molecular basis of crop responses to K^+^ deficiency.

Plant responses to K^+^ deficiency are based on a complex set of traits related to morphological, physiological, developmental, and cellular processes (7). These processes are well controlled by K^+^-responsive genes (proteins), which are linked to various cross-talk pathways. Thus, the identification of K^+^-responsive genes (proteins) is a fundamental step toward understanding their molecular mechanisms. Omics technologies allow an integrated approach to investigating nutrient deficiencies, taking into consideration all aspects of nutrient management in plants. In the past few decades, the molecular mechanisms of K^+^ deficiency have been dissected using transcriptomic approaches, and many K^+^-responsive genes have been identified. The results have been summarized previously (4–17). Although these studies have enhanced our understanding of plant responses to K^+^ deficiency, many questions remain unanswered. This is because screening for K^+^ perception mutants is hampered by the facts that K^+^-related physiological and morphological phenotypes appear late during stress and are relatively nonspecific (8), and that gene expression can be regulated at the transcriptional, post-transcriptional, translational, and post-translational levels (18).

Mass spectrometry (MS)-based proteomics offers an alternative approach to assessing changes in metabolic activity (*versus* differences in gene transcript levels), as changes in gene expression at the transcript level are not always reflected at the protein level. Therefore, proteome studies complement transcriptome analyses (19–21). Many proteins in higher plants responsive to nitrogen (N) and phosphorus (P) deficiencies have been identified *via* proteomic analysis (7, 22, 23).

However, information regarding proteins responsive to deficiencies in other essential nutrients, including K^+^, remains scarce. Proteomic studies on K^+^ deficiency to date have used two-dimensional gel electrophoresis (2-DE)^1^ and mass spectrometry analyses in a few species of higher plants (24, 25). However, only a few proteins (*e.g.* 37 and 27 K^+^-responsive proteins in *Arabidopsis* and ramie, respectively) have been identified in these studies, possibly because of the method used (limit of quantification, poor detection of some low-abundance proteins, reproducibility, etc.) (26), or the lack of a fully annotated plant genome (27). Thus, accurate, large-scale identification of proteins that respond to K^+^ deficiency and genome sequencing is needed to determine the molecular mechanism of plant K^+^ deficiency, and develop superior crop cultivars with greater K^+^ efficiency.

Bread wheat, one of the three most important cereal crops globally, feeds ∼40% of the world's population by providing 20% of the total food calories and protein in the human diet (28), and therefore, it is important to understand the molecular mechanisms of K^+^ deficiency in important food crops, because we are facing challenges to the worldwide food supply. However, bread wheat has a huge, 17-gigabase-pair (Gb), allhexaploid genome (*Triticum aestivum* L., 2n = 6x = 42, AABBDD; 2n is the number of chromosomes in each somatic cell and 6x is the basic chromosome), is rather complex, and more than 80% of the genome consists of highly repetitive sequences (29). These factors greatly impede expression analysis of the wheat transcriptome and proteome, hindering examination of the molecular mechanisms of growth and development of bread wheat compared with the other two important cereal crops, rice and maize, which have smaller genomes (∼0.4 Gb and 2.5 Gb, respectively). Isobaric tagging for relative and absolute quantification (iTRAQ), a second-generation, gel-free proteomic approach, provides more accurate quantitation and coverage at the protein level than 2-DE, particularly for low-abundance proteins (26, 30). The ordered draft genome sequence of the hexaploid wheat reference genotype Chinese Spring has recently been produced by the International Wheat Genome Sequencing Consortium (IWGSC). Survey of the gene content and composition of all 21 wheat chromosomes have identified and annotated 124, 201 gene loci (31). Thus, it is now possible to expand coverage of the bread wheat proteome and better characterize its growth, development, and responses to biotic and abiotic stresses (32). In this study, an iTRAQ-based large-scale quantitative proteomics was performed on bread wheat seedlings exposed to K^+^ deficiency. This approach was sensitive enough to delineate a specific set of K^+^-responsive protein species in bread wheat. We paid attention to protein species involved in JA synthesis, and of the JA synthesis-related protein species, an allene oxide synthase (AOS) was selected to verify the important role of JA in plant response to K^+^ deficiency using transgenic rice plants and mutants.

## EXPERIMENTAL PROCEDURES

### 

#### 

##### Plant Growth Conditions and K^+^ Deficiency Treatment

The design of this study is shown in supplemental Fig. S1. Bread wheat (*Triticum aestivum* L.) cv. Zhoumai 18 seeds were sterilized with 0.01% HgCl_2_, then washed thoroughly with distilled water. Sterilized seeds were grown hydroponically in full-strength Hoagland solution (pH 6.5) (33) in glass dishes (15 cm diameter) in a FPG-300C-30D illumination incubator (Ningbo, China) with a 14 h photoperiod, 25 °C/15 °C day/night temperatures, light intensity of 250 μmol m^2^/s, and relative humidity of 60/75% (day/night). Each dish contained about 60 seedlings. To ensure that experimental materials were sensitive to K^+^ deficiency, two-week-old wheat seedlings, which were in the autotrophic stage and sensitive to abiotic stresses (34), were used in this study. Wheat seedlings at this stage have low internal K^+^ storage capacity, exhibit a relatively high growth rate, and require high rates of K^+^ uptake from the external medium. Wheat seedlings were divided into two groups: one group remained in Hoagland medium under full K^+^ conditions (6.0 mm K^+^) as a control, and the other group was transferred to K^+^ -free (K^+^) Hoagland medium for K^+^ deficiency (KNO_3_ and KH_2_PO_4_ were replaced by NaNO_3_ and NaH_2_PO_4_, respectively) (35). No significant changes were observed in Na^+^ or Cl^−^ in the K^+^-deficiency treatment, and thus the responses of the identified proteins in the following proteomic analysis were indeed because of a change in the external K^+^ concentration rather than to changes in Cl^−^ or Na^+^ concentration, ionic strength, or osmotic potential. Hoagland solution was replaced daily during the macronutrient deficiency period. Root and leaf samples were collected at 8 d after incubation in two nutrient media, immediately frozen in liquid nitrogen, and stored at −80 °C before assessing physiological parameters or extracting total proteins. Other roots and leaves from the same sampled plants were used to determine K^+^ concentrations.

##### Growth Parameters

Growth parameters, such as plant height, and root length of wheat seedlings were determined, immediately following 10 d of K^+^ deficiency at intervals of 2 d. Dry weights of roots and leaves were determined after drying the roots and leaves for 72 h at 60 °C. The averaged values of at least four plants were considered as one replicate and three independent biological replicates were performed.

##### Determination of Phytohormones and K^+^ Concentrations

Roots and leaves were harvested separately at different sampling timepoints and dried in an oven for 72 h at 60 °C before grinding them to a fine powder. K^+^ concentrations were determined by an ICP-emission spectrometer (Perkin Elmer Optima 2100DV), as described by Ding *et al.* 2006 (36). Concentrations of some important phytohormones (JA; abscisic acid, ABA; cytokinin, CYT; and ethylene, ETH) were determined using an enzyme-linked immunosorbent assay kit (China Agricultural University, Beijing) (37–39). The average values of the above physiological parameters from roots or leaves from at least 4 plants were considered as one replicate and three independent biological replicates were performed.

##### iTRAQ Proteomic Experiment

Root and leaf proteomes were performed using three independent biological replicates with at least 6 plants in each replicate. Proteins from triplicate root or leaf samples (1.5 g) were extracted using the trichloroacetic acid/acetone method (40). Protein concentrations were determined using the Bradford protein assay (Bio-Rad, Hercules, CA) followed by our previous study (41). Total protein (200 μg) was reduced, alkylated, and digested using the filter-aided sample preparation procedure, respectively, described by our previous study (42). The digested peptide mixtures were labeled using the 8-plex iTRAQ reagent (Applied Biosystems, Foster, CA), and performed three independent 8-plex iTRAQ reagent biological replicates. Protein samples were labeled as 113–121 in each independent 8-plex iTRAQ reagent, respectively. 113-control roots, 116-K^+^-deficient roots, 117-control leaves, and 121-K^+^-deficient leaves were used in this study, and other four labels (114, 115, 118, and 119) were used other experiments. 2D LC-MS/MS analysis and protein identification were referred to supplemental Method S1 for detailed description. To substantiate our proteomic results, the genes encoding 12 K^+^-responsive protein species were randomly selected (43), and their transcription levels were measured using quantitative real-time PCR (qPCR) method. The relative transcription levels of each gene were calculated using the 2^–^^Δ^^Δ^^Ct^ method with *actin* and *phosphoglyceraldehyde dehydrogenase* (*GADPH*) genes as the two internal controls. Primers used for qPCR are listed in supplemental Table S1, and qPCR method is described in supplemental Method S2.

##### Responses of Transgenic Rice Lines Expressing TaAOS Gene to Low K^+^ or K^+^-Deficient Conditions

Coding sequence (CDS) of the gene encoding TaAOS protein was amplified using primer pairs (supplemental Table S1) and subcloned into the plasmid pCUN1301, which carries a maize (*Zea mays*) ubiquitin 1 (UBI1) promoter and a NOS terminator cassette, to construct one overexpression vector, TaAOS-OE (44). In model plant species and in crops with small genomes, functional analysis of candidate genes is often performed through chemical-, T-DNA-, or transposon-aided mutagenesis, stable transgene overexpression, or stable RNA interference (RNAi) (45). However, there are many issues in the transformation of bread wheat with large genome; *e.g.* multiple copy insertions, low transformation efficiency, high tendency for gene silencing, unstable transformation, prolonged duration, and low transformation efficiency (46–48). Rice has widely used as a model system for monocotyledonous plants and is a close relative of bread wheat (49). In this study, thus, *TaAOS* gene was transformed to rice to identify its function.

The *Agrobacterium*-mediated transformation was performed using vigorously growing calli derived from mature embryos of rice (cv. '*Nipponbare*') following a standard procedure (50). Transgenic plants expressing *TaAOS* gene were regenerated from transformed calli by selecting for hygromycin resistance, PCR analysis, and Western blot analysis as described by Wang *et al.* (51). Seedlings of two *TaAOS* transgenic rice lines (TaAOS-OE4 and 5) were grown in International Rice Research Institute (IRRI) nutrient solution (pH 5.5; 0.35 mm, K_2_SO_4_; 0.3 mm, KH_2_PO_4_) for 2 weeks in a FPG-300C-30D illumination incubator, and the solution was replaced every 2 d. The roots of some seedlings were equilibrated in the measuring solution (0.1 mm CaCl_2_ and 0.3 mm MES, pH 6.0) for 30 min. The equilibrated seedlings were then transferred to a measuring chamber filled with low K^+^ (0.3 mm, KH_2_PO_4_; K_2_SO_4_ was replaced by Na_2_SO_4_) or K^+^ deficiency (K_2_SO_4_ and KH_2_PO_4_ were replaced by Na_2_SO_4_ and NaH_2_PO_4_, respectively) for 15 d as described previously by Yang *et al.* (16). Growth parameters, K^+^ and JA concentrations of transgenic plants were also measured according to the above methods using three independent biological replicates with at least 4 plants each.

The differentially expressed 28 protein species were selected according to fold changes with random method, in which protein species were held constant but expression profiles for each protein species were assigned randomly (52). Partial cDNA sequences of their homologous rice genes were cloned from rice and the transcription levels of these genes were determined using qPCR in *TaAOS* transgenic lines suffering from K^+^ deficiency. The *18SrRNA* and *UBQ5* genes were used as two internal controls. Primers and qPCR method are listed in supplemental Table S1 and supplemental Method S2, respectively.

##### Responses of JA-Defective Rice Mutant (aos) to Low K^+^ and K^+^ Deficiency

Because it is less straightforward to produce gene mutants in polyploid species (*e.g.* bread wheat) than it is in diploid species (*e.g.* rice) and many rice mutants are available, this species has widely been employed to determine the biological function of interest genes from bread wheat (53, 54). Here, T-DNA inserted mutants of *OsAOS* [*osaos*, homozygote (PFG_1B-23433) or heterzygote (PFG_1B-23323) background *Oryza sativa* japonica *cv*. Dongjin], were kindly provided by the Crop Biotech Institute of Kyung Hee University (55, 56). Homozygote plants of these two *osaos* mutants were identified using PCR as described previously (50) and primers are indicated in supplemental Table S1. Seedlings of *osaos* homozygote mutants and wild-type (WT, Dongjin) plants were grown in IRRI nutrient solution for 2 weeks and then exposed to low K^+^ (0.3 mm) or K^+^ deficiency for 15 d. Their growth parameters were measured according to the methods as described above, and three independent biological replicates with at least 4 plants each were used. Genomic DNA was isolated and purified from 0.1 g four-week-old wild or mutant rice seedlings, following the method described previously (57).

##### Statistical Analysis

One-way analysis of variance (ANOVA) using SPSS version 17.0 statistical software and Duncan's multiple range test (DMRT), were used to identify significant differences (*p* < 0.05) among group means. Data are means ± standard deviation (S.D.) for at least three independent experiments.

## RESULTS

### 

#### 

##### Phenotypic and Physiological Changes in Wheat Seedlings Exposed to K^+^ Deficiency

**Fig. 1.**
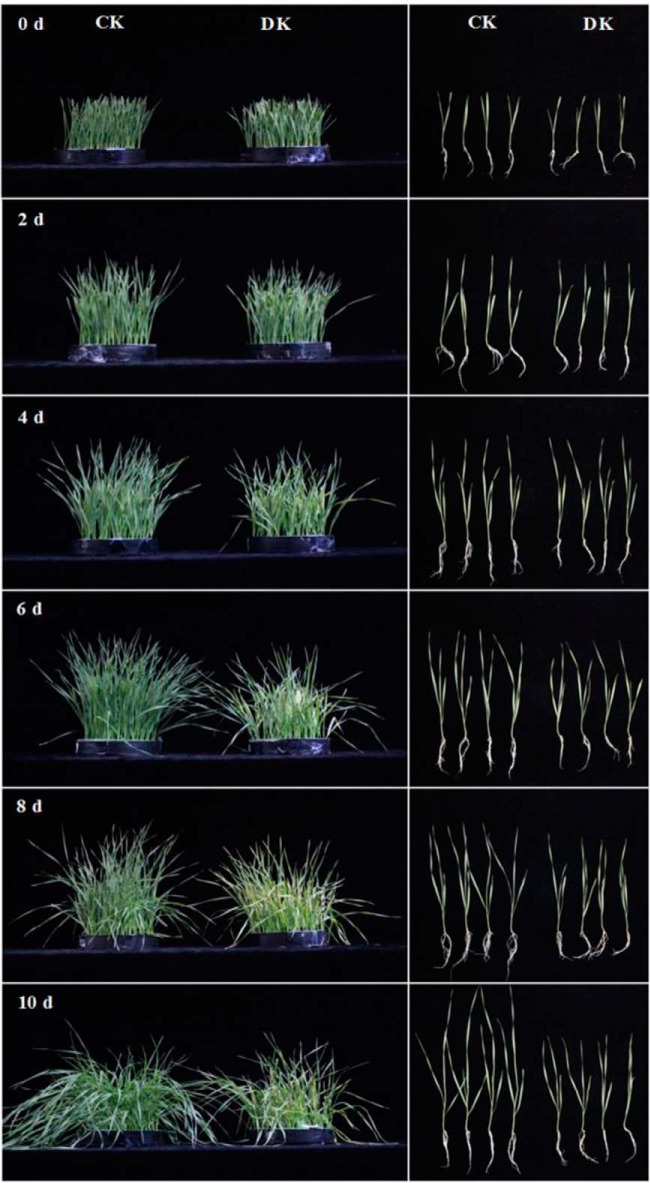
**Phenotypes of wheat seedlings under normal and K^+^-deficient conditions at indicated times.** Two-week-old wheat seedlings were divided into two groups: CK, full K^+^ conditions; DK, K^+^ deficiency (KNO_3_ and KH_2_PO_4_ were replaced by NaNO_3_ and NaH_2_PO_4_, respectively).

**Fig. 2.**
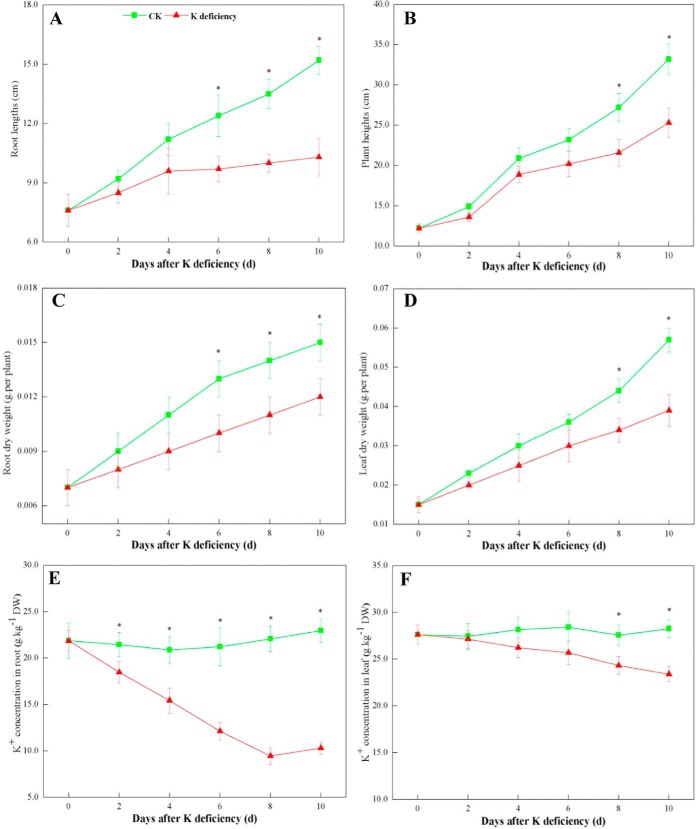
**Root length (*A*), plant height (*B*), root dry weight (*C*), leaf dry weight (*D*), root K^+^ concentration (*E*), and leaf K^+^ concentration (*F*) of wheat seedlings suffering from K^+^ deficiency for 10 d.** Each value is the mean ± standard deviation of three independent biological replicates. Asterisks indicate significant differences (*p* < 0.05).

Two-week-old wheat seedlings were transferred to K^+^-deficient full-strength Hoagland medium for 10 d. After treatment for 0, 2, 4, 6, 8, and 10 d, wheat seedlings' K^+^-deficient phenotypes were evaluated. Phenotypes of K^+^-deficient and control seedlings exhibited no differences after K^+^ deficiency treatment for 4 d. Wheat seedlings showed visible phenotypic differences at 6 d after K^+^ deficiency, and thereafter, the K^+^-deficient seedlings exhibited stunted growth, shown by smaller roots and leaves than control (Fig. 1). Measurements of root length, plant height, and dry weight of roots and leaves of K^+^-deficient wheat plants indicated marked and continuous declines in these parameters after K^+^ deficiency (Fig. 2*A*–2*D*). After 10 d of exposure to K^+^ deficiency, wheat seedlings showed significant (*p* < 0.05) decreases in root length, plant height, root dry weight, and leaf dry weights of 32.2%, 22.8%, 20.0%, and 31.6%, respectively, relative to control seedlings.

Root K^+^ concentrations markedly decreased after only 2 d, whereas leaf K^+^ concentrations declined until 8 d after K^+^ restriction; moreover, K^+^ concentrations in roots were considerably lower than those in leaves throughout K^+^ deficiency treatment (Fig. 2*E* and 2*F*). After 10 d of K^+^ deficiency, K^+^ concentrations in wheat roots decreased markedly by 74.4% (Fig. 2*E*). K^+^ concentrations in leaves remained relatively high, whereas also remarkably declining by 17.3% (Fig. 2*F*).

Generally, short-term nutrient deficiency is a result of a significant decrease in the level of the corresponding nutrient, and long-term nutrient deficiency is further characterized by the presence of a distinct phenotypic symptom and the major alteration of metabolic activities (7). Under long-term nutrient deficiency, plants exhibit more nutrient-responsive proteins (genes) than under short-term deficiency (58, 59). Identifying differentially expressed genes (proteins) in plants subjected to long-term nutrient deficiency can help to reveal the mechanisms underlying plant adaptation to mineral nutrient deficiency (7). In this study, all phenotypic parameters (root length, plant height, and root and leaf dry weights) of K^+^-deficient wheat seedlings were first observed at 8 d after K^+^ restriction (Fig. 2*A*–2*D*). Now, as expected, physiological parameters (K^+^ concentrations) showed significant changes (Fig. 2*E* and 2*F*). Thus, the 8-day time point should be regarded as the beginning of long-term K^+^ deficiency in wheat seedlings and, consequently, it was chosen for our proteomic analysis of wheat seedlings under long-term K^+^ deficiency.

##### Quantitative Proteomic Analysis Identified Many K^+^-responsive Protein Species in Bread Wheat Seedlings

To identify K^+^-responsive proteins, proteome profiles were generated using iTRAQ in both root and leaf tissues of bread wheat seedlings exposed to K^+^ deficiency for 8 d, respectively. Three independent biological replicates (three independent 8-plex iTRAQ reagents) were used for each sample for iTRAQ labeling. Mascot software, the NCBInr and IWGSC databases were then used to analyze the spectra, which were identified by MS/MS with 95% confidence of false discovery rate (FDR) ≤ 1% (60). Using the above methods, we identified 30,885 peptides from 151,654 spectra (supplemental Data S1), and 7349 protein species (supplemental Data S2), of which 4235 commonly appeared in all three biological replicates (supplemental Data S3). And the mass spectrometry proteomic data associated with this study have been deposited to the ProteomeXchange Consortium with the data set identifier PXD003570.

Ratios were used to assess fold changes in the abundance of protein species identified according to K^+^-deficient *versus* control plants, Duncan's multiple-range test was also used to identify significant differences (*p* < 0.05), and *p* values were adjusted using multiple testing corrections (61). Volcano plot was used to depict distributions on fold changes of the identified protein species for their biological significance (supplemental Fig. S2) and the related data are listed in supplemental Table S2. Furthermore, the identified protein species were filtered and analyzed using the cRAP database (62). Based on these criteria, 818 protein species with significantly altered abundances were identified in all three biological replicates of K^+^-deficient wheat seedlings, 400 and 418 of which occurred in roots and leaves, respectively (supplemental Data S4). The change in abundance of the majority of identified protein species in the roots (332/400, 83.0%) and leaves (318/418, 76.1%) of K^+^-deficient wheat seedlings was ±1.20 ∼ ±1.49 fold, whereas only 168 and 37 protein species showed fold changes between ±1.50– ∼ ±1.99 and ≥ 2.00– or ≤ −2.00 fold in these two tissues, respectively (supplemental Fig. S3, supplemental Data S5). Further, fold changes of ≥ +1.20– and ≤ −1.20-fold were significant in the abundance of regulated protein species in supplemental Data S4 (64). Some identified proteins had the same names and accession numbers but different isoelectric points (pI) or molecular weights (MW) (supplemental Data S6). These proteins should be classified into distinct protein species, because they could be different products of one gene and might have different cellular tasks, because of the presence of nucleotide polymorphisms, alternative splicing, proteolytic cleavage, and post-translational modifications (63). For instance, two globulin 3 (gi 215398470) were identified in root proteomic data, but they belong to different protein species because of their different pI and MW values (supplemental Data S6). Seventy K^+^-responsive protein species were commonly identified in both root and leaf tissues (supplemental Data S7). Other K^+^-responsive protein species accounting for the larger proportions (327/400, 81.8% in roots, and 325/418, 77.8% in leaves) differed between these two tissues (supplemental Data S4).

Transcription levels of the genes encoding the 12 K^+^-responsive protein species were measured using qPCR method with *actin* and *GADPH* genes as the two internal controls. Using the wheat *actin* gene as the internal control, we found that the transcription profiles of the 5 genes were approximately coincided with their folds in proteomic data (supplemental Fig. S4*A*, S4*C*, S4*D*, S4*K*, and S4*L*), whereas the transcription profiles of the other 7 genes were different (supplemental Fig. S4*B*, and S4*E*–S4*J*). Similar data were obtained using *GADPH* gene as the other internal control (supplemental Fig. S5).

##### Categories of the Differentially Expressed K^+^-responsive Protein Species in Both the Root and Leaf Tissues

**Fig. 3.**
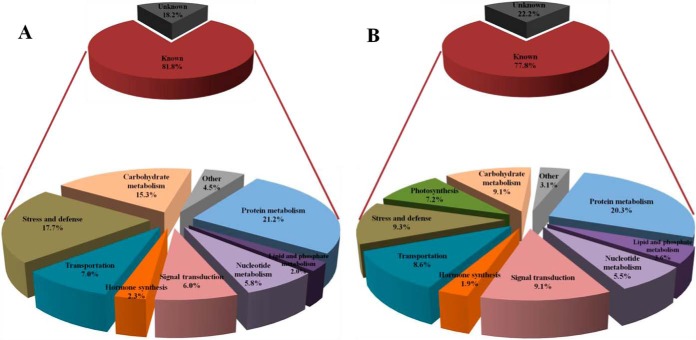
**Functional categorization of differentially expressed K^+^-responsive protein species identified in both root (*A*) and leaf (*B*) tissues of wheat seedlings suffering from K^+^ deficiency for 8 d.**

The differentially expressed protein species under K^+^-deficient conditions were functionally annotated against the NCBInr and IWGSC databases and then grouped on the basis of their biological functions. The differentially expressed protein species identified in both root and leaf tissues of K^+^-deficient wheat seedlings were then categorized into hormone synthesis, signal transduction, transportation, stress and defense, photosynthesis, carbohydrate metabolism, protein metabolism, nucleotide metabolism, lipid and phosphate metabolism, other metabolisms, and previously unknown function (Fig. 3). Predicted, hypothetical, uncharacterized, unnamed, and no special function protein species were also classified into the unknown function category. A small number of function-known protein species related to sulf, alkaloid, tetrapyrrole, etc., were assigned to other metabolisms. Many function-unknown protein species (73/400 and 18.2% in roots; 93/418 and 22.2% in leaves) were also found in this study (supplemental Data S4), possibly because of the complexity of the bread wheat genome, in which many proteins have not been characterized (29). There were some differences in the ratio of categories of K^+^-responsive protein species between root and leaf tissues of wheat seedling exposed to K^+^ deficiency. For example, the amounts and ratios of the stress and defense-related protein species (71, 17.7%) in root tissue, were more than those (39, 9.3%) in leaf tissue.

##### Hormone Synthesis-related Protein Species and JA Concentrations

**Fig. 4.**
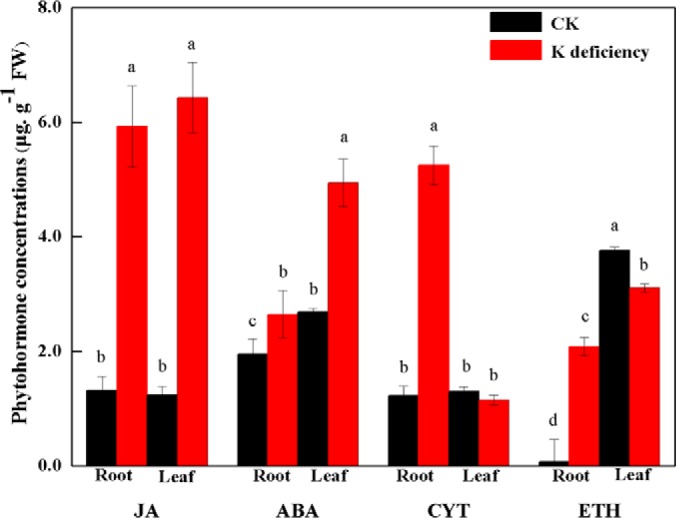
**Concentrations of the phytohormones JA, ABA, CYT, and ETH in the root and leaf tissues of wheat seedlings suffering from K^+^ deficiency for 8 d.** Each value is the mean ± standard deviation of three independent biological replicates. Different letters indicate statistically significant differences (*p* < 0.05).

Protein species with the enhanced abundance and hormones with the increased concentrations can play important roles in abiotic and biotic stresses in higher plants (62). Our proteomic analysis showed that seven, one, and one protein species were related to JA, CYT, and ETH synthesis in the root tissue of K^+^-deficient wheat seedlings. Moreover, five, one, one, and one protein species were involved in JA, ABA, CYT, and ETH synthesis in the leaf tissue, respectively, and most (8/9 in the root and 5/8 in the leaf) of these protein species were upregulated (supplemental Data S4). Concentrations of phytohormones (JA, ABA, CYT, and ETH) were next measured. Most of JA, ABA, CYT, and ETH concentrations in both root and leaf tissues of K^+^-deficient wheat seedlings also increased strongly (Fig. 4). After 8 d of K^+^ deficiency, JA concentrations in both root and leaf tissues of wheat seedlings increased by 4.50- and 5.20-fold, respectively, highest among all the checked hormones (Fig. 4).

**Fig. 5.**
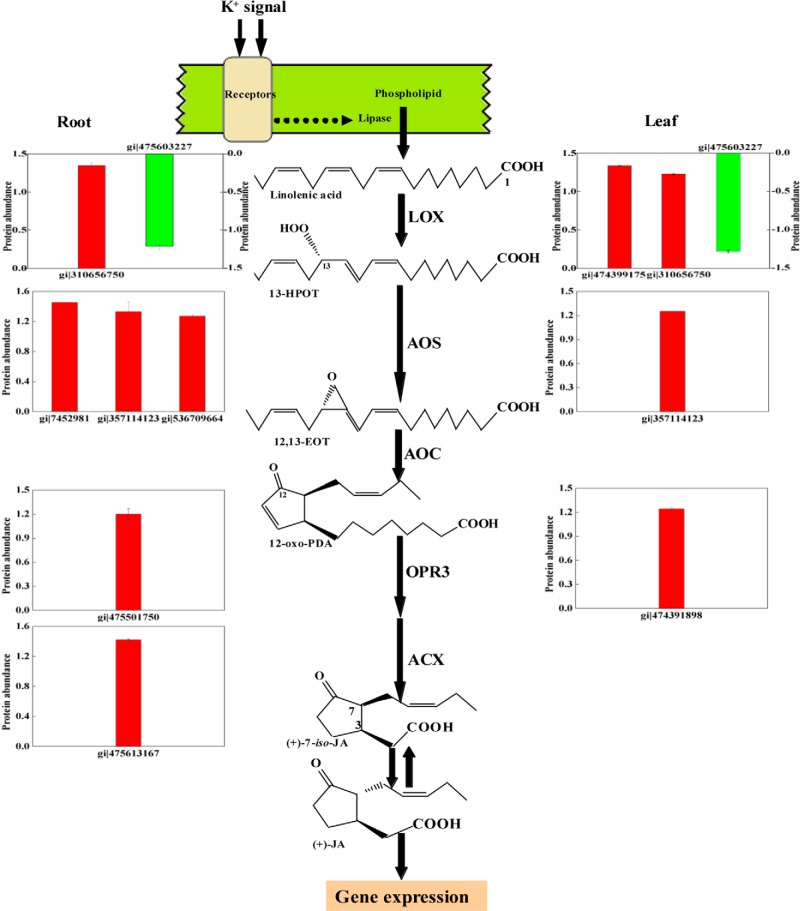
**Detection of differentially expressed K^+^-responsive protein species involved in the JA biosynthetic pathway.** It is postulated that free α-linolenic acid forms 13(*S*)-hydroperoxyoctadecatrienoic acid by the action of lipoxygenase (LOX). The resulting hydroperoxide, 13(*S*)-hydroperoxyoctadecatrienoic acid (13-HPOT) is converted into an unstable allene oxide, 12,13(*S*)-epoxy-(9*Z*,11*E*,15*Z*)- octadecatrienoic acid (12,13-EOT), by allene oxide synthase (AOS). Allene oxide cyclase (AOC) catalyzes the cyclization of 12,13-EOT to yield 12-oxo-phytodienoic acid (OPDA). OPDA is transported from the plastid into the peroxisome, where it is reduced by the action of OPDA reductase 3 (OPR3) and after three cycles of β-oxidation involved in acyl-CoA oxidase (ACX), afford (+)-*iso*-JA, which is subsequently isomerized to (-)-JA, a multifunctional protein (MFP) possessing 2-transenoyl-CoA hydratase and l-3-hydroxyacyl-CoA dehydrogenase activities, and (-)-JA then acts to modulate gene expression or can be further catabolized (78, 79). Altered abundance of the JA synthesis-related enzymes identified in this study is indicated using bar diagrams.

Most (6/7 and 85.7% in roots, 4/5 and 80% in leaves) of the differentially expressed protein species involved in JA synthesis were upregulated, including three AOSs, one lipoxygenase (LOX), one acyl-coenzyme A oxidase 3 (ACX), and one 12-oxophytodienoate reductase (OPR) in roots, and two LOXs, an AOS, and an OPR in leaves, and they are key enzymes in JA synthesis pathway (Fig. 5). To examine the role of JA in the regulation of K^+^ deficiency, we cloned the open reading frame (ORF) of the *TaAOS* gene (supplemental Fig. S6), which encodes an AOS enzyme (gi 357114123) identified in both root and leaf proteomes, and further constructed its overexpression vector (supplemental Fig. S7*A*).

##### Transgenic Rice Plants Expressing TaAOS Gene Enhanced the Tolerance to Low K^+^ or K^+^ Deficiency

**Fig. 6.**
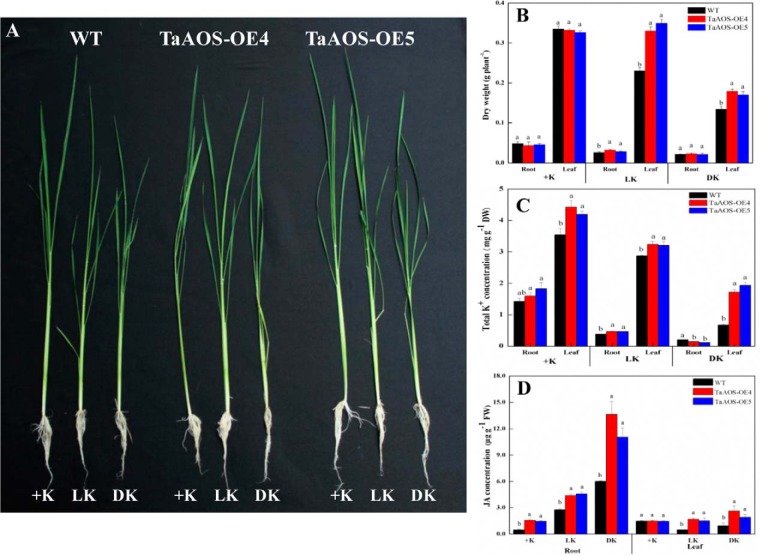
**Phenotypes, dry weights, K^+^ concentrations, and JA concentrations of two *TaAOS* rice transgenic lines (*TaAOS-OE4* and *5*) suffering from low K^+^ (0. 3 mm) or K^+^-deficient conditions for 15 d.** *A*, phenotypes of transgenic lines suffering from low K^+^ (0.3 mm) or K^+^-deficient conditions; *B*, dry weights of root and leaf tissues; *C*, total K^+^ concentrations in root and leaf tissues; *D*, JA concentrations in root and leaf tissues. +K, normal K^+^ supply. Each value is the mean ± standard deviation of three independent biological replicates. Different letters indicate statistically significant differences (*p* < 0.05). The same treatments are indicated in the following figures and supplemental data.

To further dissect the complex networks of JA-related pathways in K^+^ deficiency, in this study, *TaAOS* gene was transformed into rice (cv. “*Nipponbare*”). Based on the results of hygromycin resistance, PCR, and Western blot analyses, two *TaAOS* transgenic rice lines (TaAOS-OE4 and 5) were selected for further characterization (supplemental Fig. S7*B*–S7*E*). When compared with wild type plants (WT), these two transgenic rice lines exhibited the enhanced growth phenotypes at 15 d after low K^+^ (0.3 mm) or K^+^ deficiency (Fig. 6*A*), as quantitatively indicated by significantly increased root and leaf dry weights under low-K^+^ conditions, and the increased leaf dry weights under K^+^ deficiency (Fig. 6*B*). Moreover, total concentrations of K^+^ were measured in two *TaAOS* overexpression transgenic lines (Fig. 6*C*). Under low K^+^ conditions, K^+^ concentrations remarkably increased in both root and leaf tissues of these two transgenic rice lines (Fig. 6*C*). Under deficient K^+^ conditions, K^+^ concentrations in leaves of these two transgenic rice lines also increased significantly, whereas root K^+^ concentrations decreased compared with control wild type plants (Fig. 6*C*). At 15 d after low K^+^ and K^+^ deficiency, JA concentrations in WT roots increased and decreased by more than 3.0- and 1.5-fold in root and leaf tissues, respectively (Fig. 6*D*). Compared with WT, JA concentrations in *TaAOS* transgenic rice plants markedly increased by 1.58–∼2.27-fold in root, and 2.02–∼3.42-fold in leaf of these two *TaAOS* transgenic rice lines after 15 d of low K^+^ or K^+^ deficiency conditions, respectively (Fig. 6*D*).

**Fig. 7.**
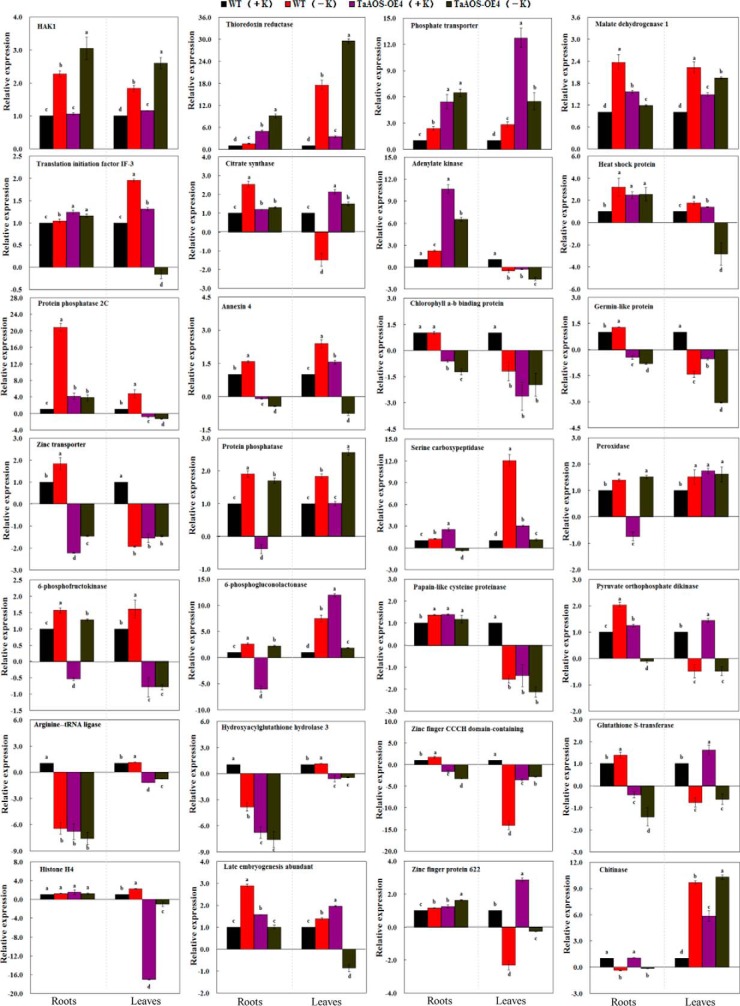
**Transcription levels of 28 genes in *TaAOS-OE4* transgenic rice lines suffering from K^+^ deficiency for 15 d.** Names of selected genes are provided in supplemental Table S1. Transcripts were determined by qPCR using the *18SrRNA* gene as the internal control. Each value is the mean ± standard deviation of at least three independent measurements. Different letters indicate statistically significant differences (*p* < 0.05).

To determine whether JA regulates the expression of the K^+^-responsive protein species, we randomly selected 28 K^+^-responsive protein species from our proteome data and further identified their homologous rice protein species in the NCBInr database. These genes encoding homologous rice protein species were isolated using rice cDNA as an amplification template (supplemental Table S1). Transcription levels of these homologous rice genes were determined by using qPCR method in both *TaAOS-OE4* and *TaAOS-OE5* transgenic rice lines grown under K^+^ deficiency for 15 d, using the rice *18SrRNA* gene as the internal control (Fig. 7; supplemental Fig. S8). Similar data were obtained using *OsUBQ5* gene as the other internal control (supplemental Figs. S9 and S10). qPCR analysis indicated that, at 15 d after K^+^ deficiency, the transcription levels of many (18/28 in root, and 14/28 in leaf) selected genes were markedly changed in the *TaAOS-OE4* transgenic rice line (Fig. 7, supplemental Fig. S9), and their transcription profiles were similar with those in our wheat proteome (supplemental Data S6). Similar results were found in the *TaAOS-OE5* transgenic rice line, as indicated in supplemental Figs. S8 and S10.

##### Rice aos Mutants Showed More Sensitivity to Low K^+^ or K^+^ Deficiency

**Fig. 8.**
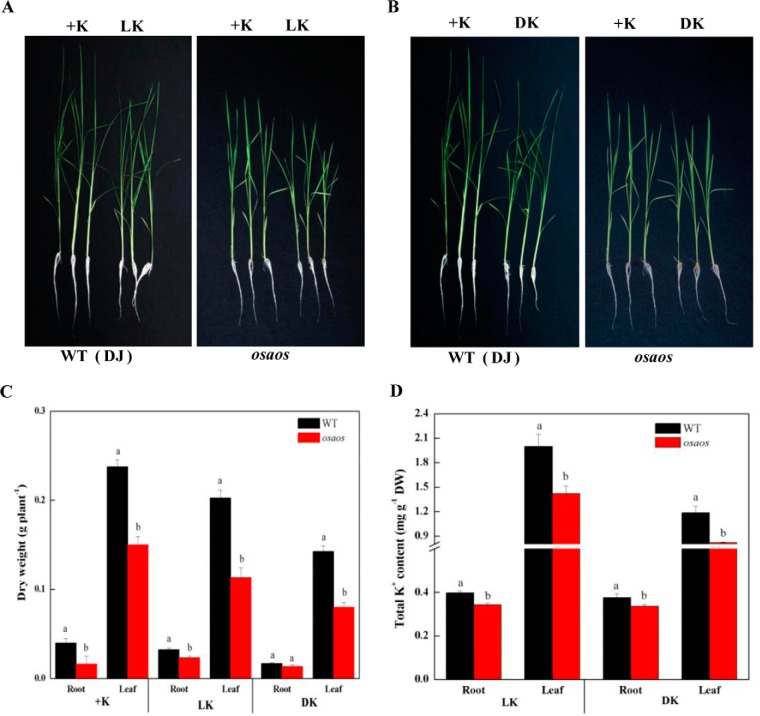
**Phenotypes, dry weights, and K^+^ concentrations of rice *osaos* mutant lines suffering from low K^+^ (0. 3 mm) or K^+^-deficient conditions for 15 d.** *A*, phenotypes of rice *osaos* mutant lines suffering from low K^+^ (LK, 0.3 mm) conditions; *B*, phenotypes of rice *osaos* mutant lines suffering from K^+^-deficient (DK) conditions; *C*, dry weights of root and leaf tissues for rice *aos* mutant lines suffering from low K^+^ (LK, 0.3 mm) or K^+^-deficient (DK) conditions; *D*, K^+^ concentrations of root and leaf tissues for rice *aos* mutant lines suffering from low K^+^ (LK, 0.3 mm) or K^+^-deficient (DK) conditions. Each value is the mean ± standard deviation of three independent biological replicates. Different letters indicate statistically significant differences (*p* < 0.05). The same treatments of +K, LK, and DK are indicated in the Fig. 6.

To study the biological function of AOS protein, a T-DNA inserted mutant of *OsAOS* (*osaos*, PFG_1B-23433), whose homozygous mutant plants were identified using PCR-based reverse screening approach (supplemental Fig. S11), was used and its K^+^ stress sensitivity was further evaluated. In this mutant, JA concentrations in both root and leaf tissues significantly decreased by 49.2% and 51.8% compared with its wild-type (WT) plants (Dongjin), respectively (supplemental Fig. S12). After 15 d of low K^+^ or K^+^ deficiency, *osaos* mutants showed more sensitive phenotypes (Fig. 8*A* and 8*B*). These phenotypic results were confirmed by quantitative analysis. Dry weights and K^+^ concentrations in leaf and root tissues of *osaos* mutants decreased by 55.9% ∼ 80.4%, and 69.3% ∼ 90.5% under low K^+^ or K^+^ deficiency conditions, respectively (Fig. 8*C* and 8*D*). Moreover, another *osaos* (PFG_1B-23323) was also used to evaluate JA function in plant response to K^+^ deficiency, and similar experimental data were indicated in supplemental Fig. S13.

## DISCUSSION

As mentioned above, several leading research groups have investigated K^+^ deficiency in plants and their results have enhanced our understanding on the mechanisms of plant tolerance to K^+^ deficiency (3–14, 16, 59, 65, 66, 68–70). In this study, we contributed to this effort by performing iTRAQ-based proteome profiling and transgenic experiments of bread wheat seedlings exposed to long-term K^+^ deficiency. These provided genome-wide protein expression profiles, which facilitated study of the translational regulation of the responses of plants to K^+^ deficiency. In discussion section, we focus on the following aspects: differences in adaptive mechanisms to deficiencies in three major nutrients (N, P, and K); differential responses between root and leaf tissues of wheat seedlings to K^+^ deficiency; comparison of our results with previous transcriptome profiles in responses to K^+^ deficiency among higher plants; and identified K^+^-responsive protein species and role of JA in plants response to K^+^ deficiency.

### 

#### 

##### Adaptive Mechanisms May Differ Among K, N, and P Deficiencies in Higher Plants

Based on their levels in plants, essential mineral nutrients are classified into macronutrients (*e.g.* N, P, and K) and micronutrients (*e.g.* iron and boron). Thus, differences in the phenotypes caused by deficiencies were compared among these three macronutrients in higher plants. Wheat seedlings showed visible phenotypic differences after long-term K^+^ deficiency (Fig. 1). Our quantitative results also showed that growth parameters (plant heights, root lengths, and leaf and root DWs) of wheat seedlings were reduced markedly at 10 d after K^+^ restriction (Fig. 2). These suggested that the growth of both root and leaf tissues of wheat seedlings was remarkably inhibited by K^+^ deficiency. It has been speculated that inhibition of root systems is likely to be a consequence of impaired biomass export because plants lacking K^+^ are less able to translocate photosynthetic products (*e.g.* sucrose, glucose, etc.) to the root *via* the phloem (59, 67). Deficiencies in N and P also inhibit leaf growth, but greatly stimulate root growth and increase the root-to-shoot ratio, suggesting that, under N and P deficiencies, plants allocate a greater proportion of their biomass to their root systems. This could facilitate the responses of plants to N and P deficiencies and more effective foraging for minerals with low availability in the rhizosphere (3, 7, 9, 65). These results reveal that higher plants use differential strategies to adapt to K, N, and P deficiencies.

K^+^ deficiency markedly decreased the K^+^ concentrations in both root and leaf tissues of wheat seedlings (Fig. 2*E* and 2*F*). However, under K^+^-deficient conditions, K^+^ concentrations in roots of wheat seedlings decreased more rapidly than those in leaves, and K^+^ concentrations in roots were markedly lower than those in leaves at 10 d after K^+^ restriction (Fig. 2*E* and 2*F*). These imply that the efficiency of K^+^ translocation from roots to leaves in wheat plants may be enhanced or K^+^ transport from leaves to roots may be impaired during K^+^ deficiency (16). Moreover, K^+^ in roots of K^+^-deficient plants was almost exhausted after long-term K^+^ deficiency, possibly resulting in the marked inhibition to root growth.

##### Differential Responses of Root and Leaf Tissues of Wheat Seedlings to K^+^ Deficiency

Based on our proteomic data, the differentially expressed 400 and 418 protein species were identified in both root and leaf tissues of wheat seedling, respectively (supplemental Data S4). These protein species belong to wide range of functional categories (Fig. 3). Some of the differentially expressed K^+^-responsive protein species were commonly identified in both root and leaf tissues of K^+^ deficient wheat seedlings, suggesting that they may have similar biological functions in the response to K^+^ deficiency in the two tissues (supplemental Data S7). However, most of differentially expressed protein species appeared specifically either in root or leaf tissues, implying that the root and leaf tissues could have differential K^+^-response mechanisms. The differences in the differentially expressed protein species between root and leaf tissues may be related to their differential functions, growth environments, and sensitivities to long-term K^+^ deficiency. The differential proteome profiles for root and leaf tissues further suggested that long-term K^+^ deficiency may have profound and different effects on growth in root and leaf tissues of wheat seedlings.

##### Comparison of Present Proteome Profiles with Previous Transcriptome Profiles among Responses to K^+^ Deficiency in Higher Plants

Transcriptome profiles during the response to K^+^ deficiency in higher plants have been reported previously (8, 11, 15, 17, 59, 67, 68). Here, a recently reported transcriptome profile of bread wheat plants responses to K^+^ deficiency was not compared with our results, because the identified K^+^-responsive genes were not provided in this literature (17). Alternatively, our root proteome profile of K^+^-deficient bread wheat seedlings was compared with the root transcriptome profile of K^+^-deficient rice seedlings published by Ma and his colleagues (59), because bread wheat and rice are close relatives, and there were many similarities between the two studies, *e.g.* similar sampling materials (whole plants), similar experimental conditions (K^+^-free solutions), the same fold change cutoff (± 1.20), the same database (NCBInr), and the same sampling timepoints (long-term K^+^ deficiency, on appearance of visible K^+^-deficient phenotypes). We found that, of the differentially expressed 339 function-known root protein species or their isoenzymes, 163 (163/339, 48.1%) were also identified by the transcriptome analysis. These common identified protein species functioned mainly in a variety of cellular processes, including transportation, stress and defense, carbohydrate metabolism, and protein metabolism (supplemental Table S3). These suggest that the concerted interplay of a diverse suite of protein species can coordinate adaptation to K^+^ deficiency in both bread wheat and rice species.

However, many detected K^+^-responsive protein species (genes) were identified only in one of these two experiments. For instance, most (6/7) of hormone synthesis-related protein species in root tissue of K^+^-deficient wheat seedlings in our proteome data were associated with JA (supplemental Data S4). In contrast, most (23/30) hormone synthesis-related genes in root tissue of K^+^-deficient rice seedlings in the transcriptome analysis were related to auxin (59). In addition, JA concentrations were significantly increased in both root and leaf tissues of K^+^-deficient wheat seedlings (Fig. 4), whereas this phytohormone was markedly increased and decreased in the root and leaf tissues of K^+^-deficient WT rice seedlings (Fig. 6*D*). These imply that different phytohormones might play differential roles among plant species or their different tissues in the response to K^+^-deficiency stress. Besides different plant species in the two studies, it was speculated that most K^+^-responsive genes identified in transcriptome analysis could be regulated post-transcriptionally, resulting in the small number of protein species commonly identified or differential expression profiles. This was also indicated by the different expression profiles of many (7/12, 58.3%) genes (protein species) at transcriptional and translational levels (supplemental Data S4, supplemental Figs. S4 and S5). Thus, the protein species identified in this study enhanced our understanding of plant responses to K^+^ deficiency.

##### Identified K^+^ -responsive Protein Species and JA Role in Plants Response to K^+^ Deficiency

Signal transduction-, stress and defense-, photosynthesis-, carbohydrate metabolism-, protein metabolism-, nucleotide metabolism-, lipid and phosphate metabolism-, other metabolism-related protein species were identified in our proteomics results (supplemental Data S4), and most of these protein species have functionally been identified and reviewed (4–17, 66–71). For instance, signaling regulatory components control the expression of suites of multiple genes, and act as activators of physiological responses to K^+^ deficiency. Phosphatases (PP2Cs and other Ser/Thr protein phosphatases) can act as counteracting molecules in protein phosphorylation pathways that signal K^+^ conditions (72–74). High-affinity K^+^ (HAK) transporters have been considered to play a crucial role when external K^+^ availability is low (16, 75). Because of their low copy numbers, however, detection of signaling regulatory components and K^+^ transporters by mass spectrometry is problematic (76). Strikingly, our proteomic data indicated that abundance of two PP2Cs (gi 475573357 and gi 475489186), and two HAKs (gi 474426623 and gi 475606875) was significantly changed in K^+^-deficient wheat seedlings (supplemental Data S4), suggesting that they could be involved in signaling transduction and K^+^ transport in plant responses to K^+^ deficiency, respectively.

Phytohormones are the foremost signaling molecules in plants, and regulate many developmental processes and adaptive stress processes because they affect several plant physiological and developmental processes (77). Previous proteomic studies on K^+^ deficiency provided few data on the regulatory protein species involved in phytohormones because the low abundance of phytohormone synthesis-related enzymes limits the detection of these protein species using 2-DE proteomic approaches (24, 25). To our knowledge, however, there is little information available on the protein species associated with hormone synthesis in previous K^+^-deficient proteomic analysis (24, 25, 68, 71). Thus, hormone synthesis-related protein species identified in our study were evaluated and their function was further confirmed using transgenic method.

In the present study, our experimental data could confirm important role of phytohormone JA in plant response to long-term K^+^ deficiency by using both proteomic and transgenic methods with the following results. Our proteomic data identified seven and five JA synthesis-related enzymes in both root and leaf tissues of K^+^-deficient wheat seedlings (Fig. 5), these comprise most of the key enzymes involved in JA synthesis (78, 79), their number was far more than that of the differentially expressed protein species involved in other hormone synthesis (supplemental Data S4), and two JA synthesis-related genes (a lipoxygenase and a jacalin-like lectin) have previously been identified in K^+^-deficient rice seedlings (11). Second, JA concentrations increased quickly in both root and leaf tissues of K^+^-deficient wheat seedlings, and were higher than those of other phytohormones (Fig. 4). Third, transgenic rice plants expressing *TaAOS*, encoding one of JA synthesis-related key gene (gi 3571141123) identified in this study, exhibited the markedly improved tolerance to low K^+^ and K^+^ deficiency by increasing K^+^ acquisition at low K^+^ condition and upward K^+^ transport from root to leaf under K^+^-deficient conditions, respectively (Fig. 6). Higher K^+^ concentrations in leaf tissue of transgenic plants under K^+^ deficiency might partially be from increased K^+^ accumulation under normal K^+^ supply condition (Fig. 6*C*) (16). Fourth, rice *osaos* mutants exhibited more sensitivity to low K^+^ and K^+^ deficiency and significantly decreased K^+^ acquisition under low K^+^ or K^+^ deficiency conditions (Fig. 8*D*). Fifth, transcription levels of the genes encoding many protein species, *e.g.* pathogenesis-related protein, chitinase, K^+^ transport protein (HAK1), and heat shock protein, were regulated significantly in the *TaAOS* transgenic rice plants after exposure to K^+^ deficiency (Fig. 7; supplemental Figs. S8–S10), their homologous proteins were identified in our wheat proteome data (supplemental Data S6), and they were also found to be JA-responsive in previous studies (80, 81).

**Fig. 9.**
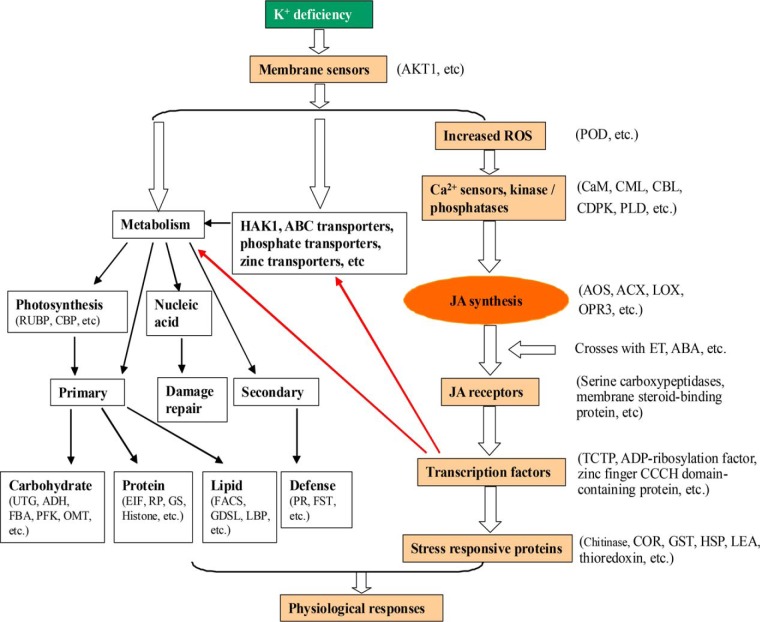
**Schematic model of the molecular responses underlying adaptation to K^+^ deficiency in higher plants.** ABA, abscisic acid; ACX, acyl-coenzyme A oxidase; ADH, alcohol dehydrogenase; AKT1, K^+^ transporter 1 (a shaker type K^+^ inward rectifying channel); AOS, allene oxide synthase; CaM, calmodulin; CBL, calcineurin B-like protein; CBP, chlorophyll a-b binding protein; CDPK, Ca^2+^-dependent protein kinase; CML, CaM-like protein; COR, cold induced protein; EIF, eukaryotic translation initiation factor; ET, ethylene; FACS, fatty acyl-CoA synthetase; FBA, fructose-bisphosphate aldolase; FST, flavonol 4′-sulfotransferase; GDSL, GDSL esterase/lipase; GS, glutamine synthetase; GST, glutathione S-transferase; HAK1, high affinity K^+^ transporter 1; HSP, heat shock protein; JA, jasmonic acid; LBP, lipid-binding protein; LEA, late embryogenesis abundant protein; LeuC, 3-isopropylmalate dehydratase; LOX, lipoxygenase; OMT, O-methyltransferase; OPR3, 12-oxophytodienoate reductase; PFK, 6-phosphofructokinase; PLD, phospholipase D; POD, peroxidase; PR, pathogenesis related protein; ROS, reactive oxygen species; RP, ribosomal protein; RUBP, Ribulose-1,5-bisphosphate carboxylase/oxygenase; TCTP, translationally controlled tumor protein; UGT, UDP-glycosyltransferase.

Based on our experimental data and previous transcriptome data (8, 11), we proposed a putative JA-dependent signaling pathway in higher plants under K^+^ deficiency (Fig. 9). In this putative JA signaling pathway, plant cells could perceive external K^+^ deficiency and generate initial K^+^ signaling, and this K^+^ signaling is subsequently transduced or encoded by ROS and Ca^2+^ sensors, which then induce translational changes in many functional protein species related to signaling components, including phytohormones (*e.g.* JA) and transcription factors. JA and transcription factors then regulate the downstream transcriptional, translational, and posttranslational responses, and finally, plants exert many morphological and physiological adaptive changes that assist survival under K^+^-deficiency stress. In our proteomic analysis, we identified a series of protein species related to these signal sensors, ROS, Ca^2+^ sensors, and transcription factors in the K^+^ signaling pathway in bread wheat (Fig. 9, supplemental Data S4), implying that they could function in perceiving and transducing K^+^ deficiency signals and function in the adaptation of bread wheat to K^+^-deficiency stress. Extensive further investigations are warranted to integrate JA and these signaling regulatory components into a comprehensive signaling network, which would greatly increase our understanding on the regulatory mechanisms of plant responses to K^+^-deficiency stress.

Similar with enhanced abundance of CYT and ETH synthesis-related enzymes, concentrations of these phytohormones also increased strongly in both root and leaf tissues of K^+^-deficient wheat seedlings (Fig. 4), implying that other phytohormones could interact with JA and play roles in the response to K^+^ deficiency in bread wheat (79). ABA concentrations also increased markedly in roots of K^+^-deficient wheat seedlings (Fig. 4), whereas ABA synthesis-related enzymes were not identified in this tissue (supplemental Data S4), possibly because of their low abundance, making detection by mass spectrometry problematic.

## Data Availability

The mass spectrometry proteomics data have been deposited to the ProteomeXchange Consortium via the PRIDE partner repository with the dataset identifier PXD003570.

## Supplementary Material

Supplemental Data

## Footnotes

^1^ The abbreviations used are:

2-DE: Two-dimensional gel electrophoresis
ABA: Abscisic acid
AOS: Allene oxide synthase
CYT: Cytokinin
ETH: Ethylene
IRRI: International Rice Research Institute
iTRAQ: Isobaric tagging for relative and absolute quantification
IWGSC: International Wheat Genome Sequencing Consortium
JA: Jasmonic acid
qPCR: Quantitative real-time PCR.
