# Supplementary material for: Large-scale Proteomics Combined with Transgenic Experiments Demonstrates An Important Role of Jasmonic Acid in Potassium Deficiency Response in Wheat and Rice
Source: Mol Cell Proteomics. 2017 Aug 18;16(11):1889–905. doi: 10.1074/mcp.RA117.000032 (PMC5671998; doi:10.1074/mcp.RA117.000032)
Supplement: Supplemental Data [file supp_16_11_1889__index.html]

Large-scale proteomics combined with transgenic experiments demonstrates an important role of jasmonic acid&nbsp;in potassium&nbsp;deficiency response in wheat and rice — JA role in responses to K+&nbsp;deficiency — Large-scale Proteomics Combined with Transgenic Experiments Demonstrates An Important Role of Jasmonic Acid in Potassium Deficiency Response in Wheat and Rice — JA Role in Responses to K+ Deficiency — Supplemental Data 

# Large-scale Proteomics Combined with Transgenic Experiments Demonstrates An Important Role of Jasmonic Acid in Potassium Deficiency Response in Wheat and Rice

## Supplemental Data

- Supplemental file 1 - Legends
- Supplemental file 2 - Figures
- Supplemental file 3-1 - Table S1
- Supplemental file 2-2 - Table S2
- Supplemental file 2-3 - Table S3
- Supplemental file 3-1 - Method S1
- Supplemental file 3-2 - Method S2
- Supplemental file 4-1 - Data S1
- Supplemental file 4-2 - Data S2
- Supplemental file 4-3 - Data S3
- Supplemental file 4-4 - Data S4
- Supplemental file 4-5 - Data S5
- Supplemental file 4-6 - Data S6
- Supplemental file 4-7 - Data S7
