# Supplementary material for: Large-scale Proteomics Combined with Transgenic Experiments Demonstrates An Important Role of Jasmonic Acid in Potassium Deficiency Response in Wheat and Rice
Source: Mol Cell Proteomics. 2017 Aug 18;16(11):1889–905. doi: 10.1074/mcp.RA117.000032 (PMC5671998; doi:10.1074/mcp.RA117.000032)
Supplement: Supplemental Data [file supp_RA117.000032_4824_0_supp_3802_3tkkw3.doc]

**Supplemental** **Information-Legends**

**Figure S1. Scheme about the experimental setup to compare K^+^-deficient wheat seedlings with unstressed wheat plants (control) using both iTRAQ proteomic and transgenic methods.**

**Figure S2. Volcano plots of the identified protein species in both root (A) and leaf (B) tissues of wheat seedlings suffering from K^+^ deficiency 8 d.**

**Figure S3. Number of differentially expressed protein species in both root and leaf tissues of wheat seedlings suffering from K^+^ deficiency for 8 d.**

**Figure S4. Transcription levels of the genes encoding 12 differentially expressed K^+^-responsive protein species identified by iTRAQ in both root and leaf tissues of wheat seedlings suffering from K^+^ deficiency 8 d (*Actin* gene as the internal control).** A, potassium transporter 1; B, zinc transporter; C, zinc finger CCCH domain-containing protein; D, allene oxide synthase; E, lipoxygenase; F, 12-oxophytodienoate reductase; G, pyruvate northophosphate dikinase; H, unnamed protein product (gi|669029255); I, chitinase; J, chlorophyll a-b binding protein; K, heat shock protein; L, alcohol dehydrogenase. Transcripts were determined by qPCR using the *Actin* gene as the internal control. All primer sequences are indicated in Table S1. Each value is the mean ± standard deviation of at least three independent measurements. Different letters indicate statistically significant differences (*P* < 0.05).

**Figure S5. Transcription levels of the genes encoding 12 differentially expressed K^+^-responsive protein species identified by iTRAQ in both root and leaf tissues of wheat seedlings suffering from K^+^ deficiency 8 d (*GAPDH* gene as the internal control).** A, potassium transporter 1; B, zinc transporter; C, zinc finger CCCH domain-containing protein; D, allene oxide synthase; E, lipoxygenase; F, 12-oxophytodienoate reductase; G, pyruvate northophosphate dikinase; H, unnamed protein product (gi|669029255); I, chitinase; J, chlorophyll a-b binding protein; K, heat shock protein; L, alcohol dehydrogenase. Transcripts were determined by qPCR using the *GAPDH* gene as the internal control. All primer sequences are indicated in Table S1. Each value is the mean ± standard deviation of at least three independent measurements. Different letters indicate statistically significant differences (*P* < 0.05).

**Figure S6. The amplified ORF sequence of *TaAOS* gene.**

**Figure S7. Molecular identification of transgenic rice lines expressing *TaAOS* gene in the background of the *Nipponbare* rice cultivar.** A, structure of *TaAOS* overexpression vector for rice transformation. The overexpression *TaAOS* vector was constructed under the control of the ubiquitin promoter, HA-flag, and nopaline synthase (Nos) terminator cassette; B, the *TaAOS* transgenic rice plants were identified by hygromycin (*Hpt II*) selection; C, the *TaAOS* transgenic rice lines were identified using PCR analysis of *Hpt II* gene; D, the *TaAOS* transgenic rice lines were further identified using PCR analysis of *TaAOS* gene. LP, RP, and LBP represent primers used for genotyping *TaAOS.* And their primer sequences are indicated in Table S1; E, western blot analysis for the *TaAOS* transgenic rice lines using an antibody against Anti-HA. Equal amount proteins are loaded to each lane and are confirmed by Anti-actin has been described by Wang *et al*. (50). All primer sequences are indicated in Table S1. Based on the above experimental results, two independent *TaAOS* transgenic rice lines (OE4 and 5) were further used for the following experiments.

**Figure S8. Transcription levels of 28 genes in *TaAOS-OE5* transgenic rice lines suffering from K^+^ deficiency for 15 d (*18SrRNA* gene as the internal control).** Names of selected genes are provided in Table S1. Transcripts were determined by qPCR using the *18SrRNA* gene as the internal control. Each value is the mean ± standard deviation of at least three independent measurements. Different letters indicate statistically significant differences (*P* < 0.05).

**Figure S9. Transcription levels of 28 genes in *TaAOS-OE4* transgenic rice lines suffering from K^+^ deficiency for 15 d (*OsUBQ5* gene as the internal control).** Names of selected genes are provided in Table S1. Transcripts were determined by qPCR using the *OsUBQ5* gene as the internal control. Each value is the mean ± standard deviation of at least three independent measurements. Different letters indicate statistically significant differences (*P* < 0.05).

**Figure S10. Transcription levels of 28 genes in *TaAOS-OE5* transgenic rice lines suffering from K^+^ deficiency for 15 d (*OsUBQ5* gene as the internal control).** Names of selected genes are provided in Table S1. Transcripts were determined by qPCR using the *OsUBQ5*gene as the internal control. Each value is the mean ± standard deviation of at least three independent measurements. Different letters indicate statistically significant differences (*P* < 0.05).

**Figure S11.** **Identification of *osaos* homozygote mutants.** A, gene structure of *OsAOS*. Black boxes indicate exons, and horizontal lines represent introns. The position of mutation in *osaos* caused by T-DNA insert is indicated with a triangle. B, *osaos* mutants were identified by PCR. RBP, LP and RP represent primers for PCR amplification and are indicated in Table S1. C, expression of *OsAOS* in the wild-type (WT, *Dongjin*) rice plants and *osaos* lines. Expression of *AOS* gene was examined by using semi-quantitative RT-PCR with 30 cycles.

**Figure S12. JA concentrations in both root and shoot tissues of *osaos* mutants.** Each value is the mean ± standard deviation of at least three independent measurements. Different letters indicate statistically significant differences (*P* < 0.05).

**Figure S13.** **T-DNA insert position of** ***osaos2* (PFG_1B-23323) and its phenotypes suffering from low K^+^ and K^+^ deficiency for 15 d.** A, The position of mutation in *osaos* caused by T-DNA insert is indicated with a triangle. Black boxes indicate exons, and horizontal lines represent introns. B, phenotypes of rice *osaos* mutant suffering from low K^+^ (LK, 0.3 mM) and K^+^-deficient (DK) conditions for 15 d.

**Method S1. 2D LC-MS/MS analysis and protein identification of iTRAQ proteomic experiment.**

**Method S2. qPCR analysis.**

**Table S1. The oligonucleotide primers of the genes used for ORF cloning, semi-quantitative PCR and qPCR analysis in this study.**

**Table S2. Data used to produce volcano plot in root (Sheet 1) and leaf (Sheet 2) tissues of wheat seedlings suffering from K^+^ deficiency for 8 d.**

**Table S3.** **Differentially expressed protein species responsive to K^+^ deficiency** **in wheat and their rice homologs at transcriptional level identified by Ma and his colleagues (ref. 59).**

**Data S1. All peptides of identified K^+^-responsive protein species in wheat seedlings suffering from K^+^ deficiency for 8 d.**

Notes: Column A, peptide sequences identified by MS/MS; Column B, peptide spectrum matching; Column C, the number of K**^+^-**responsive protein species in protein groups; Column D, the number of protein groups; Column E, modifications of peptide; Column F, the number of mass positively charged peptides.

**Data S2. All K^+^-responsive protein species identified in wheat seedlings suffering from K^+^ deficiency for 8 d.**

Notes: Asterisk and purple color represent the fold changes of ≥ +1.20- and ≤ –1.20-fold and hormone synthesis-related proteins listed in Data S4, respectively; Column A, accession numbers of K**^+^-**responsive protein species in NCBI database; Column B, names of K**^+^-**responsive protein species in NCBI database; Column C, accession numbers of K**^+^-**responsive protein species in IWGSC database; Column D, description of K**^+^-**responsive protein species in IWGSC database; Columns E, H, and K, ratios of K**^+^-**responsive protein species in treatment/control in three independent biological replicates in root; Columns F, I, and L, peptide counts of K**^+^-**responsive protein species in three independent biological replicates in root; Columns G, J, and M, varability of K**^+^-**responsive protein species of treatment/control in three independent biological replicates in root; Column N, average ratios of K**^+^-**responsive protein species of three independent biological replicates in root; Column O, significance A of K**^+^-**responsive protein species calculated according to an outlier significance score for log protein ratios in three independent biological replicates in root; Column P, *t*-test analysis of K**^+^-**responsive protein species of three independent biological replicates in root; Column Q, FDR value of K^+^-responsive protein species calculated according to BH test of three independent biological replicates in root; Columns R, U, and X, ratios of K^+^-responsive protein species of treatment/control in three independent biological replicates in leaf; Columns S, V, and Y, peptide counts of K^+^-responsive protein species in three independent biological replicates in leaf; Columns T, W, and Z, varability of K^+^-responsive protein species of treatment/control in three independent biological replicates in leaf; Column AA, average ratios of K^+^-responsive protein species in three independent biological replicates in leaf; Column AB, significance A of K^+^-responsive protein species calculated according to an outlier significance score for log protein ratios in three independent biological replicates in leaf; Column AC, *t*-test analysis of K^+^-responsive protein species in three independent biological replicates in leaf; Column AD, FDR value of K^+^-responsive protein species calculated according to BH test in three independent biological replicates in leaf; Column AE, the number of amino acids of K^+^-responsive protein species; Column AF, theoretical molecular weight of K^+^-responsive protein species; Column AG, theoretical calculation of the isoelectric points (pI) of K^+^-responsive protein species; Column AH, coverage of K^+^-responsive protein species; Column AI, the number of K^+^-responsive protein species; Column AJ, unique peptides of K^+^-responsive protein species; Column AK, the number of peptides of K^+^-responsive protein species; Column AL, peptide spectrum matching of K^+^-responsive protein species; Column AL, protein scores of K^+^-responsive protein species; The blank panes (rows) imply no value (ratio, etc) in one of three independent biological replicates.

**Data S3. All K^+^-responsive protein species identified in all three independent biological experiments in wheat seedlings suffering from K^+^ deficiency for 8 d.**

Notes: Asterisk and purple color represent the fold changes of ≥ +1.20- and ≤ –1.20-fold and hormone synthesis-related protein species listed in Data S4, respectively; Column A, accession numbers of K^+^-responsive protein species in NCBI database; Column B, names of K^+^-responsive protein species in NCBI database; Column C, accession numbers of K^+^-responsive protein species in IWGSC database; Column D, description of K^+^-responsive protein species in IWGSC database; Columns E-G, three independent biological replicates of K^+^-responsive protein species in root; Column H, average ratios of K^+^-responsive protein species in three independent biological replicates in root; Column I, significance A of K^+^-responsive protein species calculated according to an outlier significance score for log protein ratios in three independent biological replicates in root; Column J, *t*-test analysis of K^+^-responsive protein species in three independent biological replicates in root; Column K, FDR values of K^+^-responsive protein species calculated according to BH test in three independent biological replicates in root; Columns L-N, three independent biological replicates of K^+^-responsive protein species in leaf; Column O, average ratios of K^+^-responsive protein species in three independent biological replicates in leaf; Column P, significance A of K^+^-responsive protein species calculated according to an outlier significance score for log protein ratios in three independent biological replicates in leaf; Column Q, *t*-test analysis of K^+^-responsive protein species in three independent biological replicates in leaf; Column R, FDR value of K^+^-responsive protein species calculated according to BH test of three independent biological replicates in leaf; Column S, the number of amino acids of K^+^-responsive protein species; Column T, theoretical molecular weight of K^+^-responsive protein species; Column U, theoretical calculation of the isoelectric point (pI) of K^+^-responsive protein species; Column V, coverage of K^+^-responsive protein species; Column W, the number of K^+^-responsive protein species; Column X, unique peptides of K^+^-responsive protein species; Column Y, the number of peptides of K^+^-responsive protein species; Column Z, peptide spectrum matching of K^+^-responsive protein species; Column AA, protein scores of K^+^-responsive protein species.

**Data S4. Identified K^+^-responsive protein species with ≥ +1.20- or ≤–1.20-fold changes in both root (Sheet 1, root-1.20) and leaf (Sheet 2, leaf-1.20) tissues of wheat seedlings suffering from K^+^ deficiency for 8 d.**

Notes: + and -, upregulated and downregulated abundance of the differentially expressed K**^+^-**responsive protein species, respectively; Column A, accession numbers of K**^+^-**responsive protein species in NCBI database; Column B, names of K**^+^-**responsive protein species accession in NCBI database; Column C, plant species of K**^+^-**responsive protein species in NCBI database; Column D, functional categories of K**^+^-**responsive protein species in NCBI database; Column E, accession numbers of K**^+^-**responsive protein species in IWGSC database; Column F, description of K**^+^-**responsive protein species in IWGSC database; Column G, functional categories of K**^+^-**responsive protein species in IWGSC database; Columns H-J, three independent biological replicates in root (Sheet 1) and leaf (Sheet 2) of K**^+^-**responsive protein species; Column K, average ratios of K**^+^-**responsive protein species in three independent biological replicates in root; Column L, *t*-test analysis of K**^+^-**responsive protein species in three independent biological replicates in root; Column M, FDR values of K**^+^-**responsive protein species calculated according to BH test in three independent biological replicates in root; Column N, coverage of K**^+^-**responsive protein species; Column O, the number of K**^+^-**responsive protein species; Column P, unique peptides of K**^+^-**responsive protein species; Column Q, the number of peptides of K**^+^-**responsive protein species; Column R, peptide spectrum matching of K**^+^-**responsive protein species; Column S, the number of amino acids of K**^+^-**responsive protein species; Column T, theoretical molecular weight of K**^+^-**responsive protein species; Column U, theoretical calculation of the isoelectric points (pI) of K**^+^-**responsive protein species.

**Data S5. Detailed fold changes of K^+^-responsive protein species in root (Sheet 1, ±1.20 ~±1.49-fold; Sheet 2, ±1.50 ~±1.99-fold; Sheet 3, ≥ +2.00- or ≤–2.00-fold) and leaf (Sheet 4, ±1.20 ~±1.49-fold; Sheet 5, ±1.50 ~±1.99-fold; Sheet 6, ≥ +2.00- or ≤–2.00-fold) tissues of wheat seedlings suffering from K^+^ deficiency for 8 d.**

Notes of all columns are same as those in Data S4.

**Data S6.** **Differentially expressed K^+^-responsive protein species with the same names and different characteristic parameters.**

Notes: + and -, upregulated and downregulated abundance of the differentially expressed K^+^-responsive protein species, respectively; Column A, accession numbers of K**^+^-**responsive protein species in NCBI database; Column B, names of K**^+^-**responsive protein species in NCBI database; Column C, identified K**^+^-**responsive protein species in NCBI database; Column D, accession numbers of K**^+^-**responsive protein species in IWGSC database; Column E, names of K**^+^-**responsive protein species in IWGSC database; Columns F-H, three independent biological replicates of K**^+^-**responsive protein species in root or leaf; Column I, average ratios of K**^+^-**responsive protein species in three independent biological replicates in root or leaf; Column J, *t*-test analysis of K**^+^-**responsive protein species in three independent biological replicates in root or leaf; Column K, coverage of K**^+^-**responsive protein species; Column L, the number of K**^+^-**responsive protein species; Column M, unique peptides of K**^+^-**responsive protein species; Column N, the number of peptides of K**^+^-**responsive protein species; Column O, peptide spectrum matching of K**^+^-**responsive protein species; Column P, the number of amino acids of K**^+^-**responsive protein species; Column Q, theoretical molecular weight of  K**^+^-**responsive protein species; Column R, theoretical calculation of the isoelectric points (pI) of K**^+^-**responsive protein species.

**Data S7.** **Differentially expressed K^+^-responsive protein species commonly identified in both root and leaf tissues of wheat seedlings suffering from K^+^ deficiency.**

Notes: + and -, upregulated and downregulated abundance of the differentially expressed K^+^-responsive protein species, respectively; Column A, accession numbers of K^+^-responsive protein species in NCBI database; Column B, names of K^+^-responsive protein species in NCBI database; Column C, identified K^+^-responsive protein species in NCBI database; Column D, accession numbers of K^+^-responsive protein species in IWGSC database; Column E, names of K**^+^-**responsive protein species in IWGSC database; Column F, average ratios in three independent biological replicates of K^+^-responsive protein species in root; Column G, average ratios in three independent biological replicates of K^+^-responsive protein species in leaf.
