# Supplementary material for: Large-scale Proteomics Combined with Transgenic Experiments Demonstrates An Important Role of Jasmonic Acid in Potassium Deficiency Response in Wheat and Rice
Source: Mol Cell Proteomics. 2017 Aug 18;16(11):1889–905. doi: 10.1074/mcp.RA117.000032 (PMC5671998; doi:10.1074/mcp.RA117.000032)
Supplement: Supplemental Data [file supp_RA117.000032_4824_0_supp_3803_ptkkw3.doc]

Wheat seedlings were grown in full-strength Hoagland’s solution for two weeks

Wheat seedlings grown in full-strength Hoagland’s solution were suffered from K^+^ deficient for 8 d

Wheat seedlings continued to be grown in full-strength Hoagland’s solution for 8 d

Some plants

Some plants

The others

The others

Growth and physiological parameters

Extracted proteins from the uppermost leaves

Extracted proteins from the uppermost leaves

Growth and physiological parameters

Morphology and physiology

iTRAQ method

The identified target protein species and phytohormone JA

Schematic model of higher plants response to K^+^ deficiency

Functional analysis of *TaAOS* gene (the key enzyme of JA synthesis) in response to K^+^ deficiency

Transgenic rice lines overexpressing *TaAOS*

Important roles of JA in wheat response to K^+^ deficiency

Rice *aos* mutants

**Figure S1.** **Scheme about the experimental setup to compare K^+^-deficient wheat seedlings with unstressed wheat plants (control) using both iTRAQ and transgenic methods.**


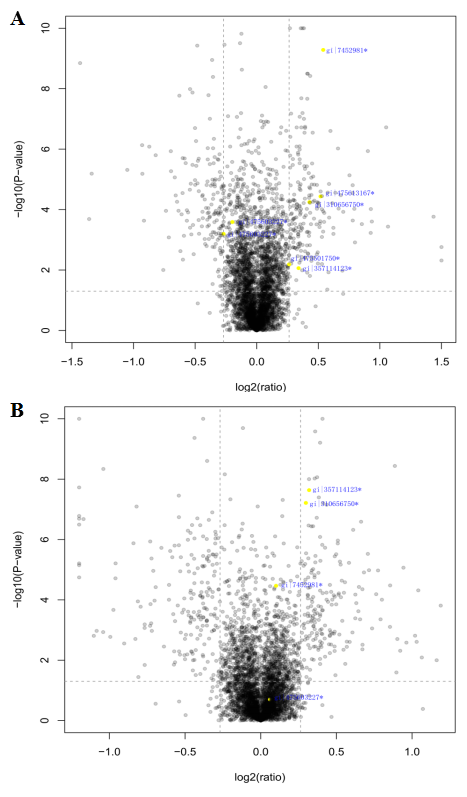


**Figure S2. Volcano plots of the identified protein species in both root (A) and leaf (b) tissues of wheat seedlings suffering from K^+^ deficiency 8 d.** The yellow dots represent the identified JA synthesis-related protein species.,gi|7452981, gi|536709664 and gi|357114123, AOS. gi|475613167, ACX; gi|310656750 and gi|475603227, LOX; gi|475501750, OPDA.


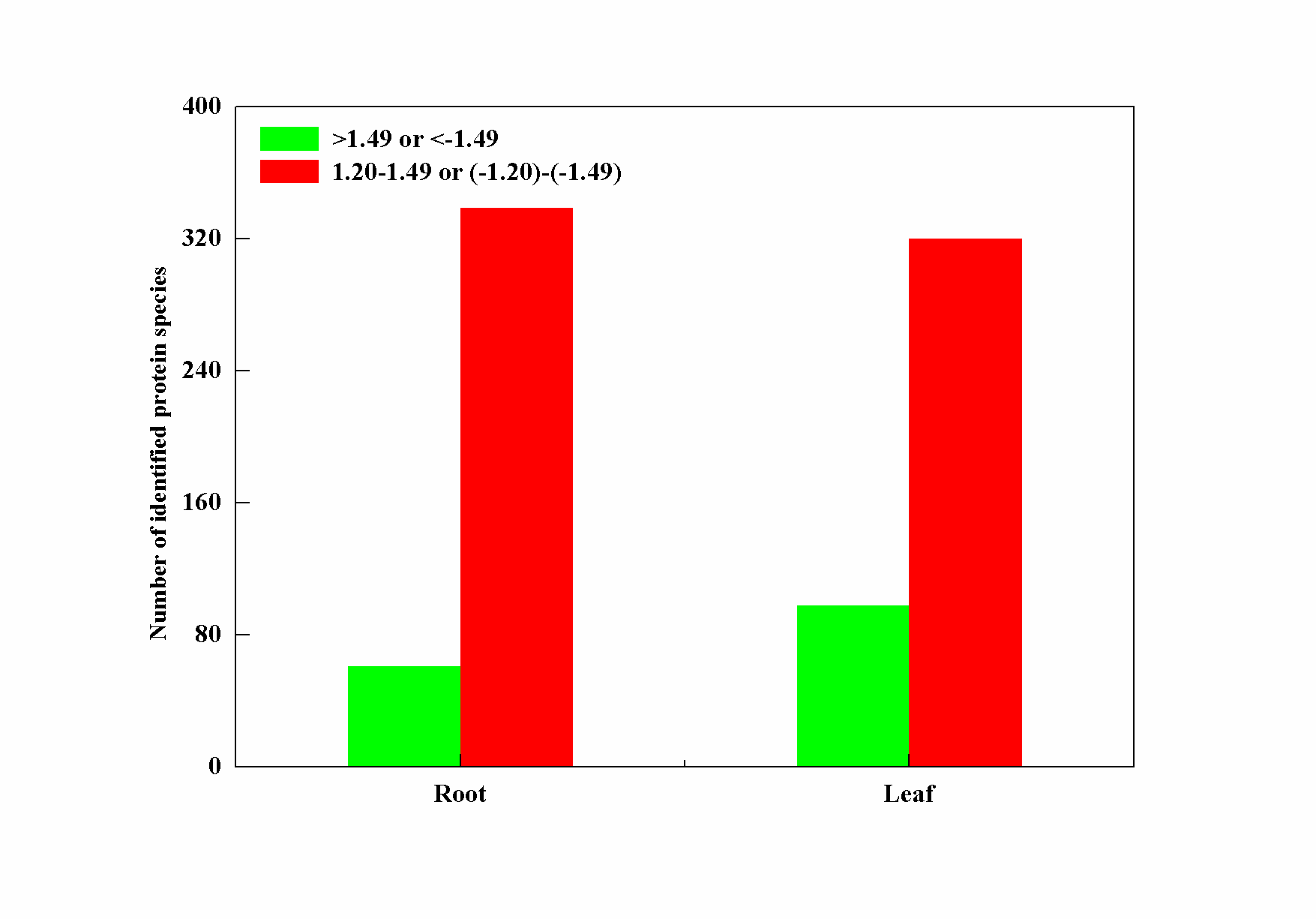


**Figure S3. Numbers of differentially expressed protein species in both root and leaf tissues of wheat seedlings suffering from K^+^ deficiency 8 d.**


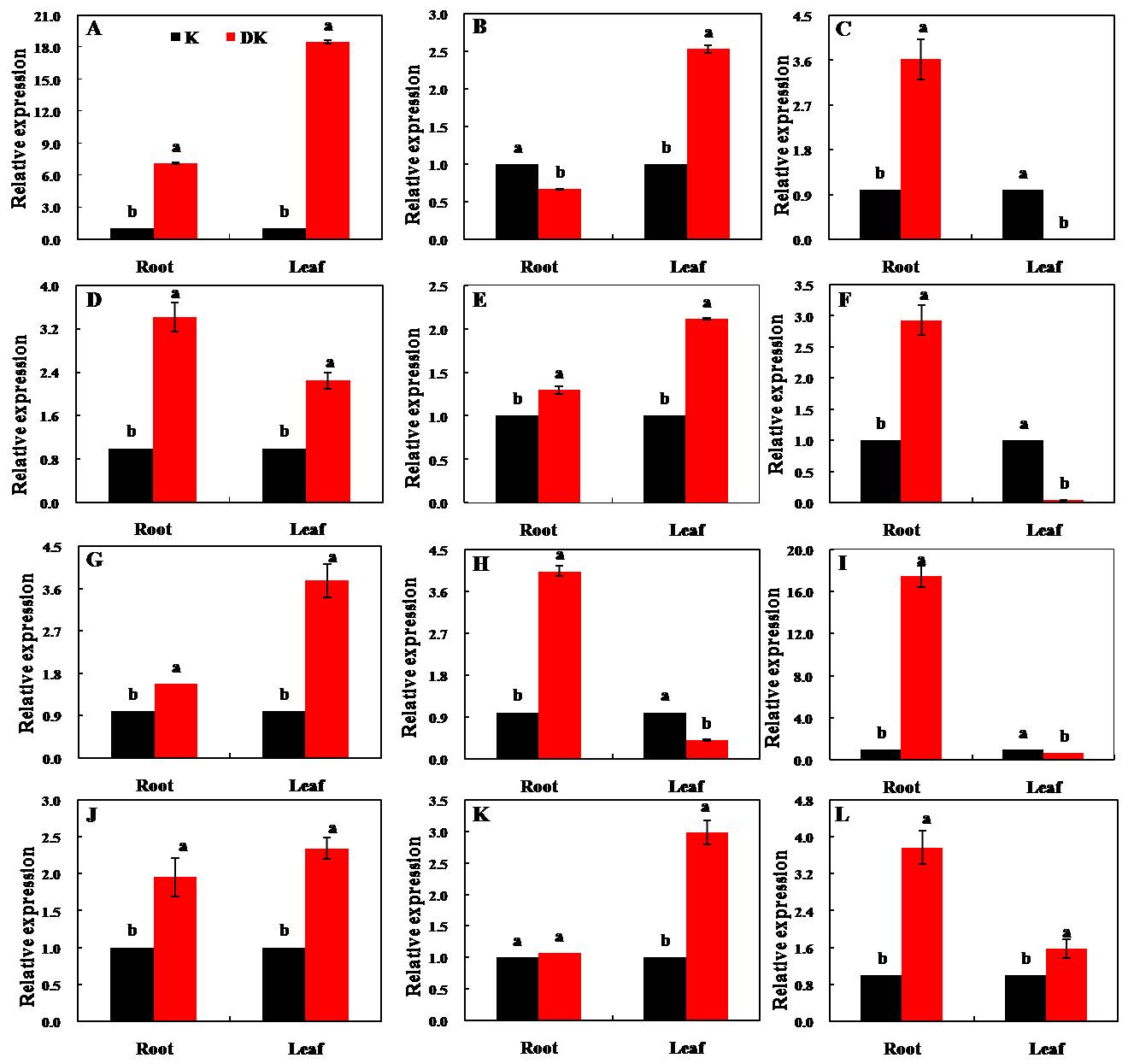


**Figure S4. Transcription levels of the genes encoding 12 differentially expressed K^+^-responsive protein species identified by iTRAQ in both root and leaf tissues of wheat seedlings suffering from K^+^ deficiency 8 d (*Actin* gene as the internal control).** A, potassium transporter 1; B, zinc transporter; C, zinc finger CCCH domain-containing protein; D, allene oxide synthase; E, lipoxygenase; F, 12-oxophytodienoate reductase; G, pyruvate northophosphate dikinase; H, unnamed protein product (gi|669029255); I, chitinase; J, chlorophyll a-b binding protein; K, heat shock protein; L, alcohol dehydrogenase. Transcripts were determined by qPCR using the *Actin* gene as the internal control. All primer sequences are indicated in Table S1. Each value is the mean ± standard deviation of at least three independent measurements. Different letters indicate statistically significant differences (*P* < 0.05).


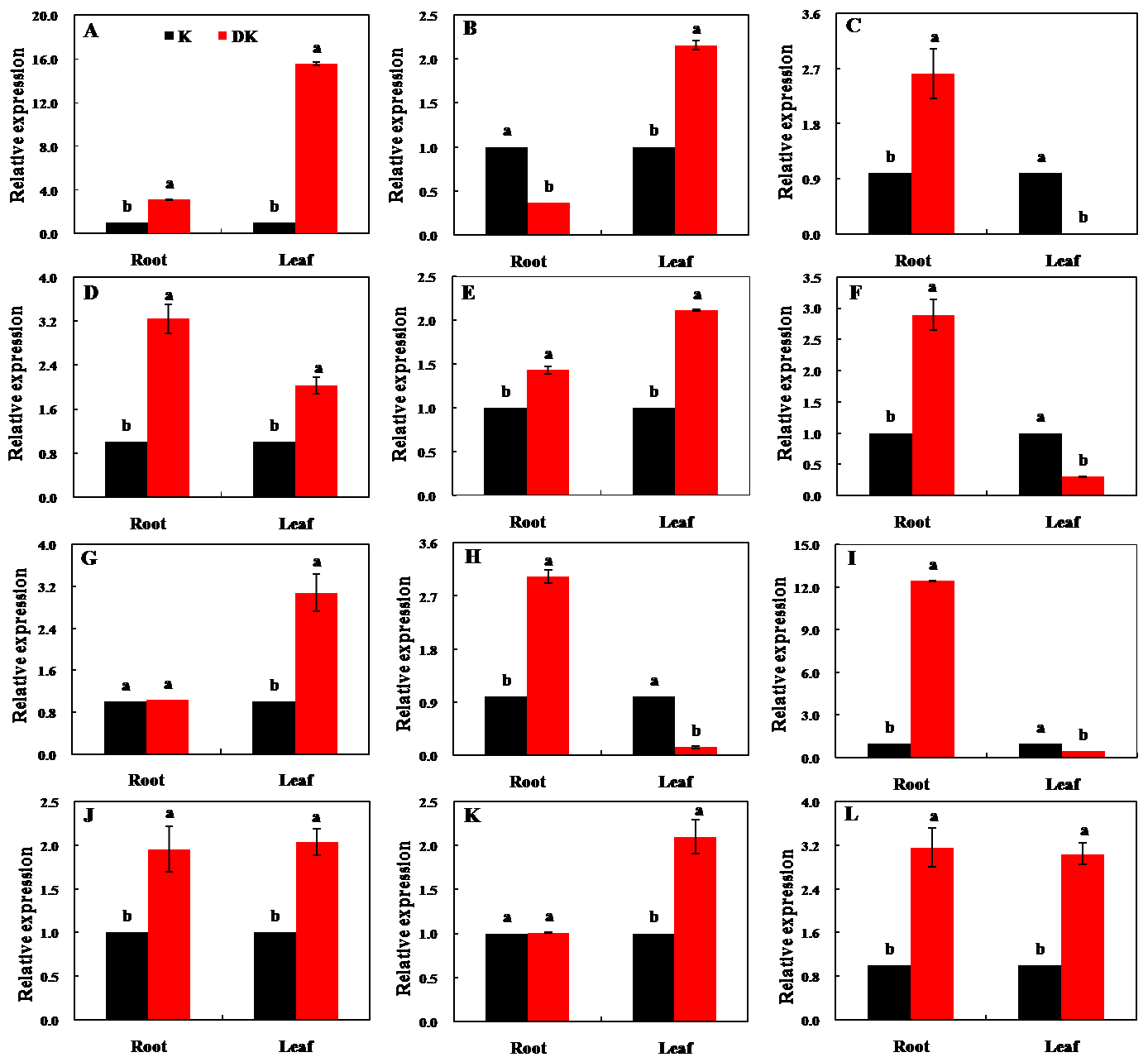


**Figure S5. Transcription levels of the genes encoding 12 differentially expressed K^+^-responsive protein species identified by iTRAQ in both root and leaf tissues of wheat seedlings suffering from K^+^ deficiency 8 d (*GAPDH* gene as the internal control).** A, potassium transporter 1; B, zinc transporter; C, zinc finger CCCH domain-containing protein; D, allene oxide synthase; E, lipoxygenase; F, 12-oxophytodienoate reductase; G, pyruvate northophosphate dikinase; H, unnamed protein product (gi|669029255); I, chitinase; J, chlorophyll a-b binding protein; K, heat shock protein; L, alcohol dehydrogenase. Transcripts were determined by qPCR using the *GAPDH* gene as the internal control. All primer sequences are indicated in Table S1. Each value is the mean ± standard deviation of at least three independent measurements. Different letters indicate statistically significant differences (*P* < 0.05).

ATGGCGGGCGGCGACGAGGGCTCCCTGGTGCCGAGGCAGGTGCCGGGCAGCTACGGCATGCCGTTCGTCTCGGCCATCCGCGACCGCCTCGACTTCTACTACTTCCAGGGCCAGGACAAGTACTTCGAGTCCCGTGTCGAGAAGTACGGCTCCACCGTCGTCCGCATCAACGTCCCGCCGGGCCCCTTCATGGCGCGCGACCCGCGGGTGGTCGCCGTGCTCGACGCCAAGAGCTTCCCCGTGCTCTTCGACGTCGACAAGGTCGAGAAGAAGAACCTCTTCACCGGCACCTACATGCCCTCCACCTCCCTCACCGGAGGCTTCCGCGTCTGCTCCTACCTCGACCCCTCCGAGCCCATCCACACCAAGGTCAAGCAGCTGCTCTTCTCCCTCCTTGCCTCCCGCAAGGACGCCTTCATCCCGGCCTTCCGTTCCCACTTCTCCTCGCTCCTCGCCACCGTGGAGTCGCAGATCGTGCTCGGCGGCAAGTCCAACTTCAACACGCTCAACGACGCCACCTCCTTCGAGTTCATCGGCGACGCCTACTTCGGCGTGCTCCCTTCTGCGTCAGACCTAGGTACCACCGGCCCGACCAAGGCCGCAAAGTGGCTCATATTCCAGCTCCACCCGCTCGTCACGCTCGGCCTCCCCATGATCCTCGAGGAGCCGCTCCTCCACACGGTGCACCTCCCTCCCATCCTCGTCAGCGGCGACTACAAGGCGCTCTACAAGTACTTCTTCGCCGCTGCGACCAAGGCGCTCGACACCGCCGAGGGCCTCGGACTGAAGCGGGACGAGGCATGCCACAACCTGTTGTTCGCCACCGTGTTCAACAGCTACGGTGGCCTCAAGGTGCTTCTCCCGGGGATCCTCGCGCGCATCGCGGGGGCCGGAGAGAAGTTCCACCAGAAGCTCGTCGCGGAGATACGCGCCGCCGTGGCGGACGCCGGCGGCAAGGTGACGATAGAGGCGCTGGAGAAGATGGAGCTGACCAAGTCGGCGGTGTGGGAGGCGCTGCGGCTGGACCCGCCCGTCAAGTTCCAGTACGGCCGCGCCAAGGCGGACATGAACATCGAGAGCCACGACGCGGTGTTCGCCGTGAAGAAGGGGGAGATGCTGTTCGGGTACCAGCCGTGCGCCACCAGGGACCCCCGCGTGTTCGGCTCCACGGCGAGGGAGTTCGTCGGCGACCGGTTCGTCGGGGAGGAGGGAAGGAAGCTGCTGCAGTACGTGTACTGGTCCAACGGGCGGGAGACCGAGAGCCCCAGCGTGGACAACAAGCAGTGCCCAGGCAAGAACCTGGTCGTGCTCGTGGGCAGGCTCCTGGTGGTGGAGCTGTTCCTCCGGTACGACACCTTCACCGCCGACGTCGGGGTCGACCTGCTCGGCACCAAGGTTGAGTTCACCGGCGTCACCAAGGCCACGTCCGGTCCTGAGAGCGCTGTTTAA

**Figure S6. The amplified ORF sequence of *TaAOS* gene.**

**
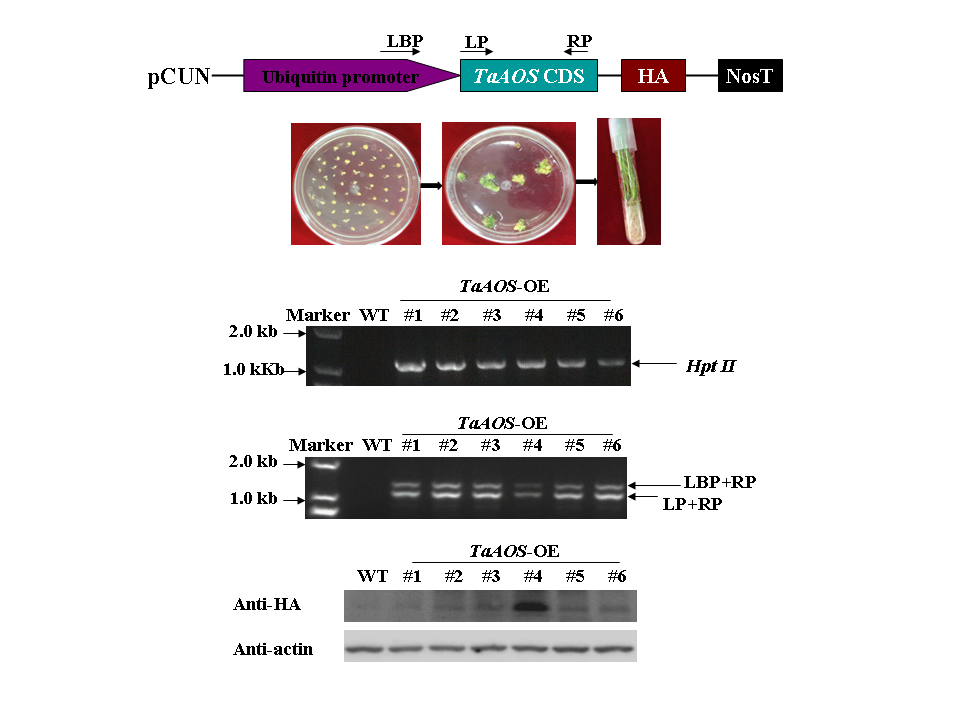
**

**B**

**C**

**D**

**E**

**A**

**Figure S7. Molecular identification of transgenic rice lines expressing the *TaAOS* gene in the background of the *Nipponbare* rice cultivar.** A, structure of *TaAOS* overexpression vector for rice transformation. The overexpression *TaAOS* vector was constructed under the control of the ubiquitin promoter, HA-flag, and nopaline synthase (Nos) terminator cassette; B, the *TaAOS* transgenic rice plants were identified by hygromycin (*Hpt II*) selection; C, the *TaAOS* transgenic rice lines were identified using PCR analysis of *Hpt II* gene; D, the *TaAOS* transgenic rice lines were further identified using PCR analysis of *TaAOS* gene. LP, RP, and LBP represent primers used for genotyping *TaAOS.* And their primer sequences are indicated in Table S1; E, western blot analysis for the *TaAOS* transgenic rice lines using an antibody against Anti-HA. Equal amount proteins are loaded to each lane and are confirmed by Anti-actin has been described by Wang *et al*. (50). All primer sequences are indicated in Table S1. Based on the above experimental results, two independent *TaAOS* transgenic rice lines (OE4 and 5) were further used for the following experiments.

**Figure S8. Transcription levels of 28 genes in *TaAOS-OE5* transgenic rice lines suffering from K^+^ deficiency for 15 d (*18SrRNA* gene as the internal control).** Names of selected genes are provided in Table S1. Transcripts were determined by qPCR using the *18SrRNA* gene as the internal control. Each value is the mean ± standard deviation of at least three independent measurements. Different letters indicate statistically significant differences (*P*< 0.05).


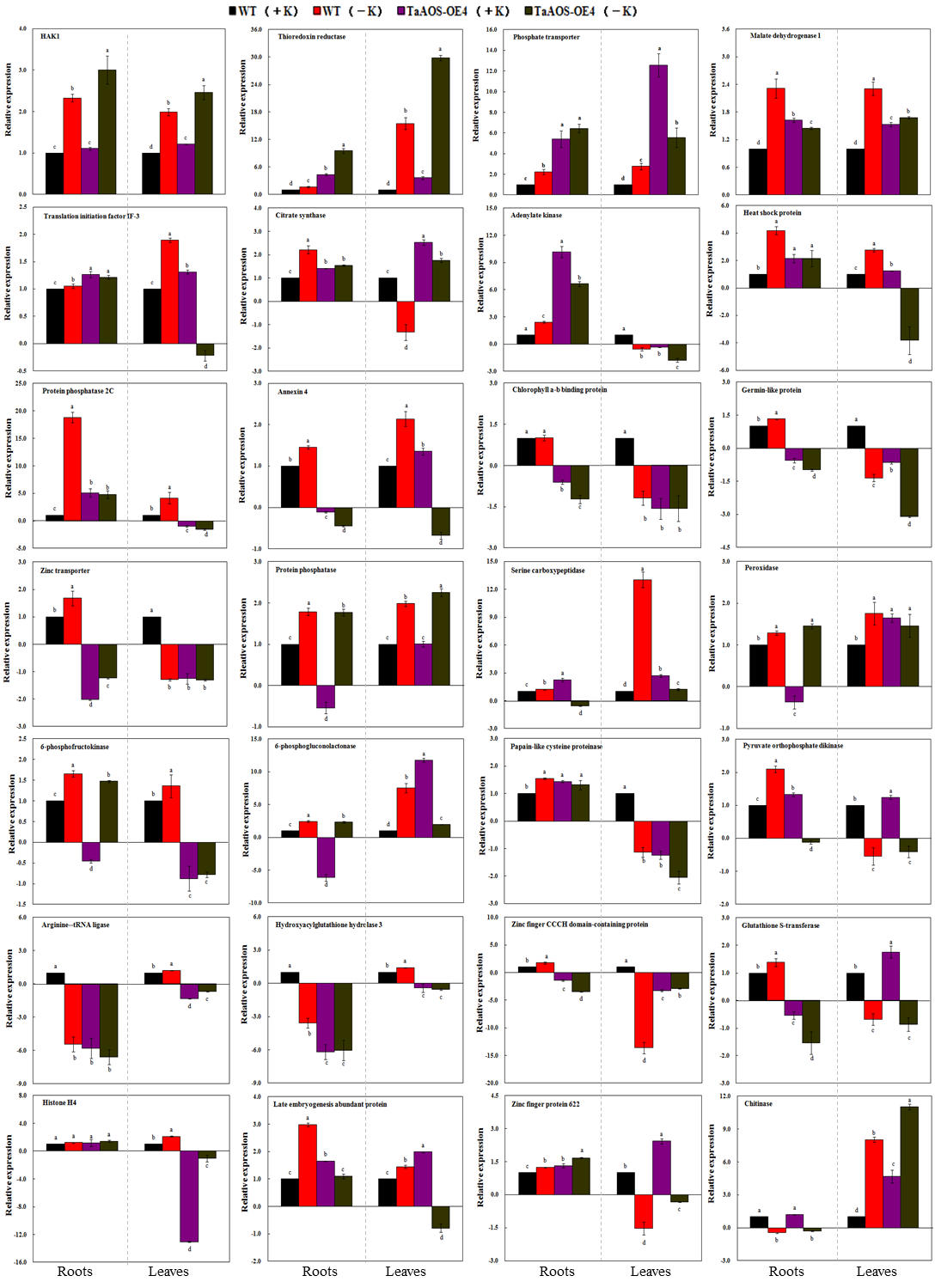


**Figure S9. Transcription levels of 28 genes in *TaAOS-OE4* transgenic rice lines suffering from K^+^ deficiency for 15 d (*OsUBQ5* gene as the internal control).** Names of selected genes are provided in Table S1. Transcripts were determined by qPCR using the *OsUBQ5* gene as the internal control. Each value is the mean ± standard deviation of at least three independent measurements. Different letters indicate statistically significant differences (*P* < 0.05).


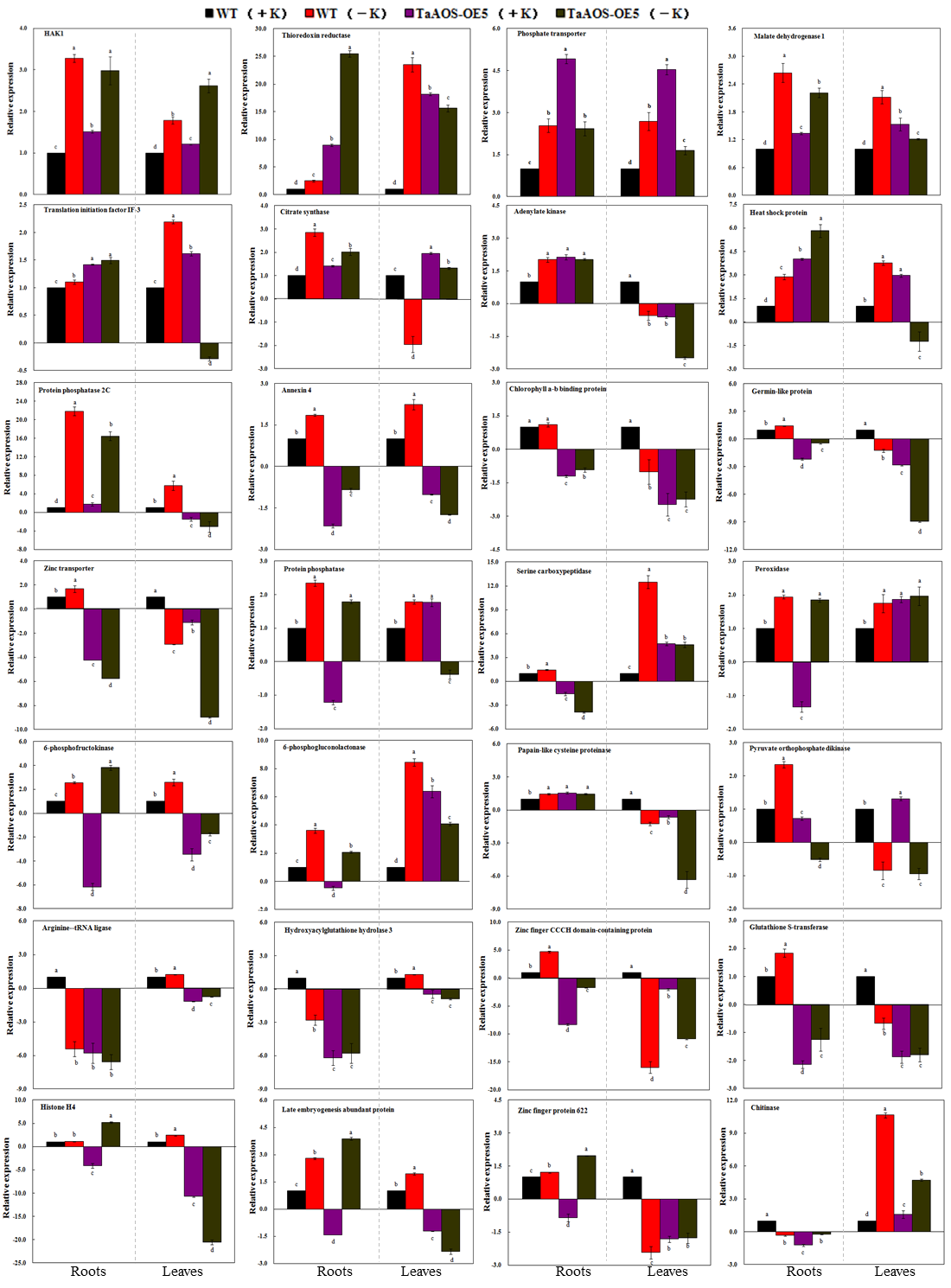


**Figure S10. Transcription levels of 28 genes in *TaAOS-OE5* transgenic rice lines suffering from K^+^ deficiency for 15 d (*OsUBQ5* gene as the internal control).** Names of selected genes are provided in Table S1. Transcripts were determined by qPCR using the *OsUBQ5* gene as the internal control. Each value is the mean ± standard deviation of at least three independent measurements. Different letters indicate statistically significant differences (*P* < 0.05).

**B**

**C**

**A**

**Figure S11.** **Identification of *osaos* homozygote mutants.** A, gene structure of *OsAOS*. Black boxes indicate exons, and horizontal lines represent introns. The position of mutation in *osaos* caused by T-DNA insert is indicated with a triangle. B, *osaos* mutants were identified by PCR. RBP, LP and RP represent primers for PCR amplification and are indicated in Table S1. C, expression of *OsAOS* in the wild-type (WT, *Dongjin*) rice plants and *osaos* lines. Expression of *AOS* gene was examined by using semi-quantitative RT-PCR with 30 cycles.


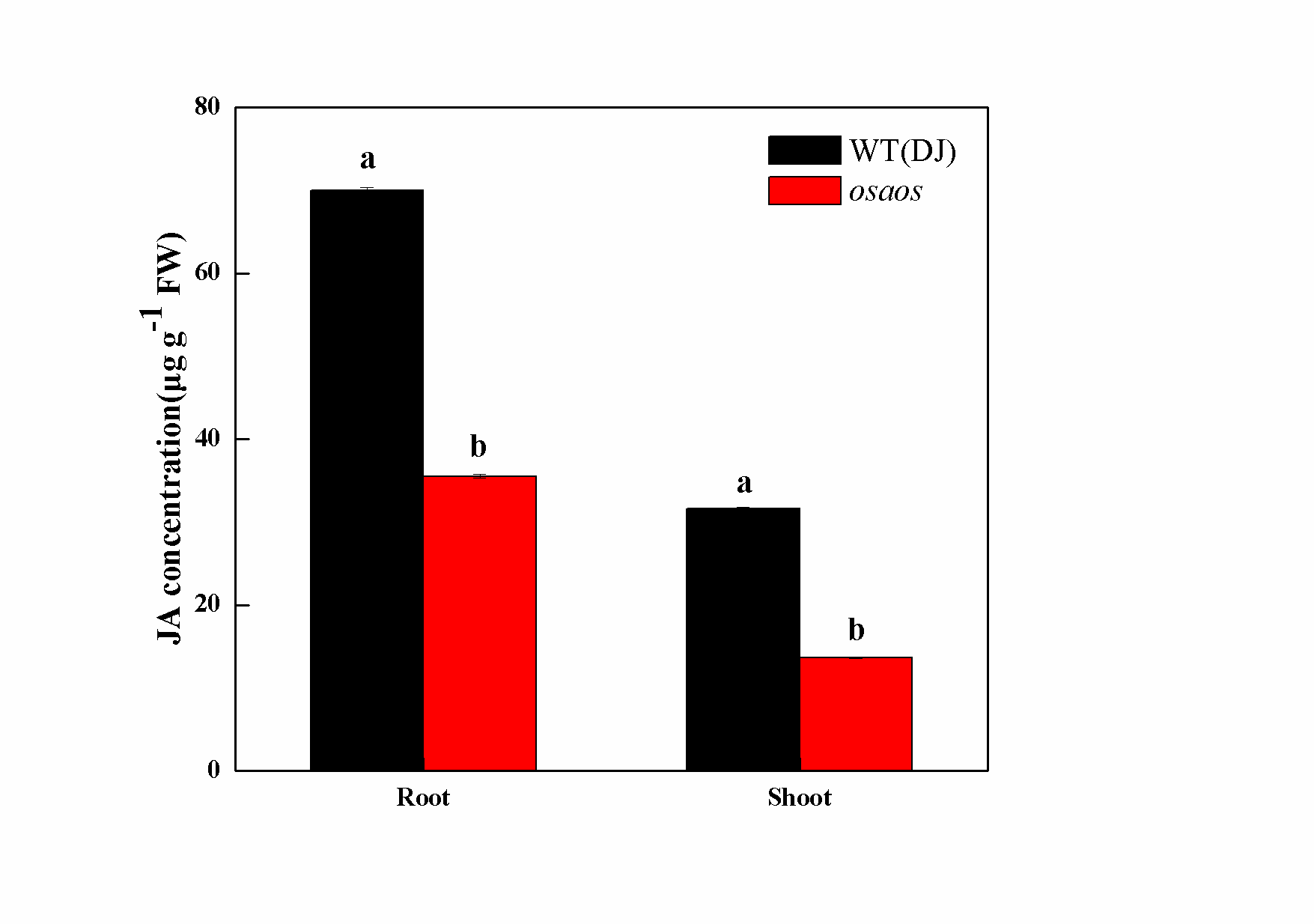


**Figure S12. ja concentrations in both root and shoot tissues of *osaos* mutants.** Each value is the mean ± standard deviation of at least three independent measurements. Different letters indicate statistically significant differences (*P* < 0.05).

**
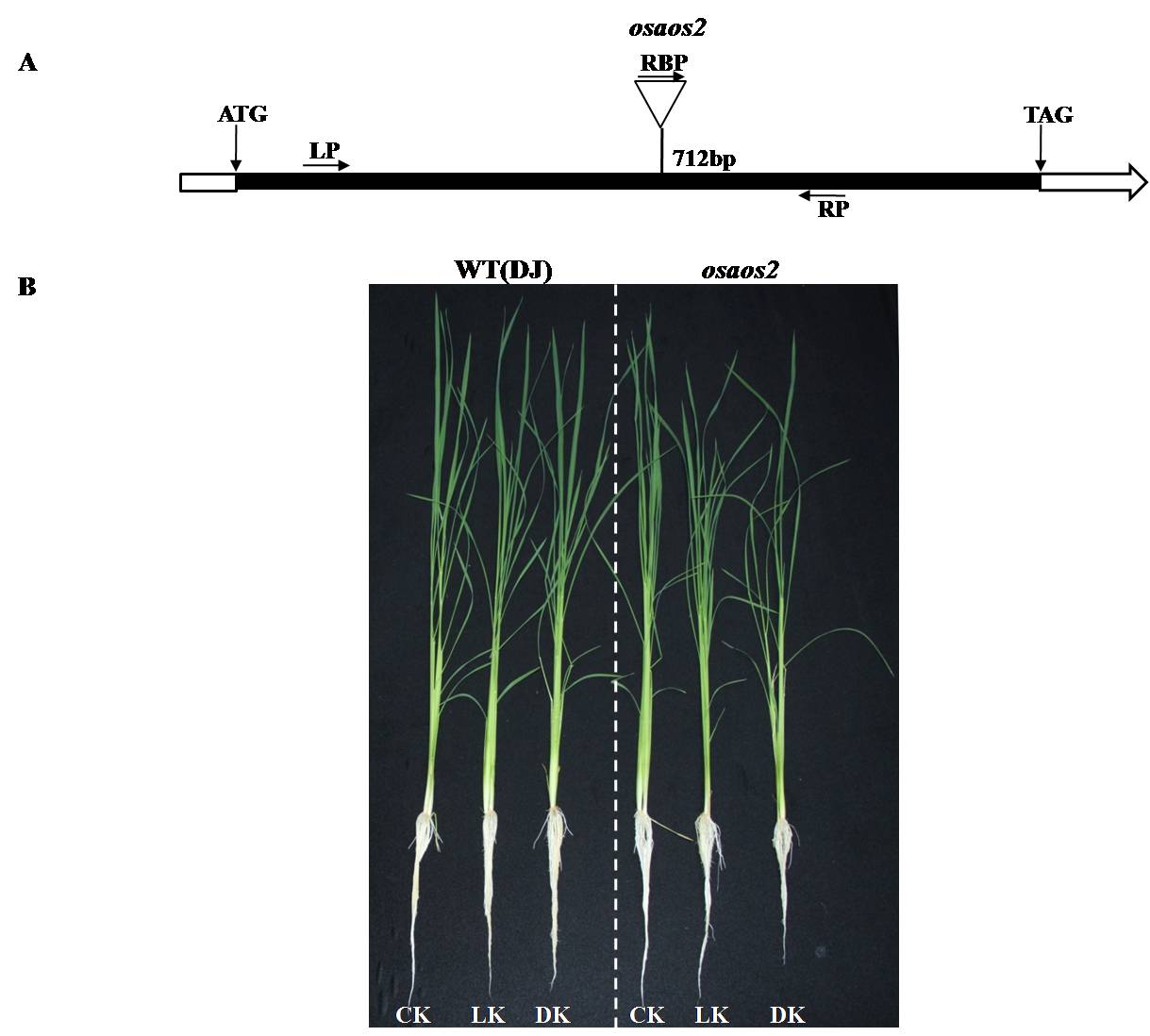
**

**Figure S13.** **T-DNA insert position of** ***osaos2* (PFG_1B-23323) and its phenotypes suffering from low K^+^ and K^+^ deficiency for 15 d.** A, The position of mutation in *osaos* caused by T-DNA insert is indicated with a triangle. Black boxes indicate exons, and horizontal lines represent introns. B, phenotypes of rice *osaos* mutant suffering from low K^+^ (LK, 0.3 mM) and K^+^-deficient (DK) conditions for 15 d.
