# Supplementary material for: Large-scale Proteomics Combined with Transgenic Experiments Demonstrates An Important Role of Jasmonic Acid in Potassium Deficiency Response in Wheat and Rice
Source: Mol Cell Proteomics. 2017 Aug 18;16(11):1889–905. doi: 10.1074/mcp.RA117.000032 (PMC5671998; doi:10.1074/mcp.RA117.000032)
Supplement: Supplemental Data [file supp_RA117.000032_4824_0_supp_3804_stkkw3.doc]

**Table S1.** The oligonucleotide primers of the genes used for ORF cloning, semi-quantitative PCR and qPCR analysis in this study.

| Types of amplification | Names of protein species | Accession no. of the identified protein species | | | Accession no. of the genes encoding the identified protein species | Primer pairs | Amplified sizes (bp) | |
| --- | --- | --- | --- | --- | --- | --- | --- | --- |
| qPCR (wheat) | Potassium transporter 1 | gi|474426623 | | | JF495466.1 | F: 5’-GGTGTTCTACGGGCCTTCAA-3’  R: 5’-GAGCTGAACAGCCCTGATGT-3’ | | 159 |
|  |  |  |  |  |  |  |  |  |
|  | Zinc transporter | gi|95114386 | | | DQ490133.1 | F: 5’- GCAGACTGGACATACGCCAT-3’  R: 5’- GAGCACACCGAGGAACTTGA-3’ | | 144 |
|  | Zinc finger domain-containing protein | gi|357123849 | | | XM_003563572 | F: 5’- CCCGAGAGAATCGGAGTCATT-3’  R: 5’- GGCCTCCAGGGAAGTTATGG-3’ | | 128 |
|  | Allene oxide synthase | gi|536709664 | | | KF039886 | F: 5’-CGCCCGTCAAGTTCCAGTA-3’  R: 5’-TGCAGCAGCTTCCTTCCCT-3’ | | 179 |
|  | Lipoxygenase | gi|310656750 | | | KC679302 | F: 5’- TTCCTGCTCAAGACGGTCAC-3’  R: 5’- GGAGAGGAAACAAGGGAGGC-3’ | | 154 |
|  | 12-oxophytodienoate reductase 1 | gi|475501750 | | | KF035083 | F: 5’- GAGGAGGCTGACGGTTGA-3’  R: 5’- GTTGGTTGTCCAGGGAAG-3’ | | 160 |
|  | Pyruvate northophosphate dikinase | gi|32400838 | | | AF475130 | F: 5’- CTTTCCCCACCAGCCCTTAG-3’  R: 5’- CCCCATTTTTCCTTGCTGCC -3’ | | 127 |
|  | Unnamed protein | | gi|669029255 | HG670306 | | F: 5’- TTGTTGATGGTTTGCAGGCG -3’  R: 5’- GAACCTGTTTCCGTTGTCGC -3’ | | 130 |
|  | Chitinase | | gi\|73622088 | KC342667 | | F: 5’- TGTTCTGGGGACGGAACAAG-3’  R: 5’- GGAGAGGTCGAGGTGGTACT-3’ | | 128 |
|  | Chlorophyll a-b binding protein | | gi\|474060351 | HM362991 | | F: 5’- CGGAGCTCAAGGTGAAGGAG-3’  R: 5’- CAGGCGTTGTTGTTGACGG-3’ | | 145 |
|  | Heat shock protein | | gi\|11561808 | AF083344 | | F: 5’- TGTTCAACACGCTCCTCCAG-3’  R: 5’- TCATGGAGTTCTTGCCCACC-3’ | | 150 |
|  | Alcohol dehydrogenase | | gi\|119388715 | EF122843.1 | | F: 5’- AAGATCCTCTACACCGCCCT-3’  R: 5’- GTTCGGTAACACCCTCTCCG-3’ | | 136 |
| **Table S1.** (*Continued*) | | | | | | | | |
| Types of amplification | Names of protein species | | Accession no. of the identified protein species | Accession no. of the genes encoding the identified protein species | | Primer pairs | | Amplified sizes (bp) |
| Internal control gene ( wheat) | Actin |  | | | AB181991 | F: 5’- AGCGGTCGAACAACTGGTA -3’  R: 5’- AAACGAAGGATAGCATGAGGAAGC -3’ | | 101 |
|  | GAPDH |  | | | EU022331 | F: 5’- TTTTCACCGACAAGGACA -3’  R: 5’- AAGAGGAGCAAGGCAGTT -3’ | | 112 |
| CDS | TaAOS | gi|7452981 | | | AJ251304 | F: 5’-ATGGCGGGCGGCGACGAG-3’  r: 5’-TTAAACAGCGCTCTCAGGACC-3’ | | 1449 |
| PCR analysis for transgenic rice lines | Hygromycin (*Hpt II*) |  | | | KT184677 | f: 5’-GAAAAAGCCTGAACTCACCGC-3’  r: 5’-TGCTCCATACAAGCCAACCAC-3’ | | 1087 |
|  | TaAOS | gi\|7452981 | | | AJ251304 | f: 5’-ATGGCGGGCGGCGACGAG-3’ (LP)  r: 5’-CCACGAGCACGACCAGG-3’ (RP) | | 1200 |
|  | TaAOS | gi\|7452981 | | | AJ251304 | f: 5’- AAGATGCCTCTGCCGACAGT-3’ (LBP)  r: 5’-CCACGAGCACGACCAGG-3’ (RP) | | 1500 |
| PCR analysis for mutant | Vector (2715) |  | | |  | RBP: 5’-CCACAGTTTTCGCGATCCAGACTG-3’ | |  |
|  | *osaos* |  | | |  | LP: 5’-GCCTTGTAGTCGGAGCTGAT-3’ | |  |
|  | *osaos* |  | | |  | RP: 5’-CCCCCACGTACATAGCACTT-3’ | |  |
| qPCR (rice) | OsHAK1 | gi|474426623 | | | AJ427970 | f: 5’-GAAGAGAGGGCAAAGTGCCT-3’  r: 5’-GCACACGCCGTTTACACAAA-3’ | | 126 |
|  | Phosphate transporter | gi\|730129899 | | | AK101170 | f: 5’-AAGAAGGCAAAAGAAATGT-3’  r: 5’-TTCGTGCCAAATTGCTGGTC-3’ | | 107 |
|  | Zinc transporter | gi\|95114386 | | | NM_001052075 | f: 5’-TCTTCAATTCCTGCGCCCAT-3’  R: 5’-CATGAACTGTTTCGGCCACG-3’ | | 126 |
|  | Protein phosphatase | gi|475573357 | | | AK065949 | F: 5’-ACGAGCTGGAACGAATCGAG-3’  R: 5’-GCTTAAGGTACCCATCCCCG-3’ | | 111 |
| **Table S1.** (*Continued*) | | | | | | | | |
| Types of amplification | Names of protein species | Accession no. of the identified protein species | | | Accession no. of the genes encoding the identified protein species | Primer pairs | | Amplified sizes (bp) |
| qPCR (rice) | Protein phosphatase | gi|475573357 | | | AK065949 | F: 5’-ACGAGCTGGAACGAATCGAG-3’  R: 5’-GCTTAAGGTACCCATCCCCG-3’ | | 111 |
|  | Zinc finger CCCH domain-containing protein | gi|357123849 | | | AB028133 | F: 5’-ACAGCTTGCAAGAACATGAGT-3’  R: 5’-GCATGATGACCTTGCCGAAC-3’ | | 103 |
|  | Histone H4 | gi|195618010 | | | AK064000 | F: 5’-TGGCAGACGATCTTGGTTCC-3’  R: 5’-ATCCGTGGCATAAGAGCTGG-3’ | | 146 |
|  | Chitinase | gi|73622088 | | | AB026998 | F: 5’-CAGAGCTCGGACATCCAGAC-3’  R: 5’-TTGTTCCACAGGTAGTCGGC-3’ | | 108 |
|  | Homeobox-leucine zipper protein | gi\|475594847 | | | U25283 | F: 5’-CTGATGGATCCGATGGACCG-3’  R: 5’-GCGACTCGAGCTCAGCAATA-3’ | | 123 |
|  | Germin-like protein | gi\|474126257 | | | AF032976 | F: 5’-TTGATTGGCTCCAGGCTCAG-3’  R: 5’-GCAAGCACACATAATTAAGCCG-3’ | | 146 |
|  | Papain-like cysteine proteinase | gi\|194352766 | | | AK105881 | F: 5’-ACTGGGATTCCCTCTTTGTTTC-3’  R: 5’-GTTCCATAGACACGATCGACCA-3’ | | 114 |
|  | Hydroxyacylglutathione hydrolase 3 | gi\|475616749 | | | AK103161 | F: 5’-CGGTCGAGCCAGATAACGAA-3’  R: 5’-TCTGGCAGATCAACACGCAT-3’ | | 139 |
|  | Arginine--tRNA ligase | gi\|357134510 | | | AK071544 | F: 5’-CACCGGCATGTTCCTGAGAT-3’  R: 5’-CAGTCACTCCAGGTGCCATT-3’ | | 142 |
|  | Chlorophyll a-b binding protein | gi\|474060351 | | | AF094776 | F: 5’-GTTTCTGCGTCCAACAGTCG-3’  R: 5’-GTGCCAAGCTCAAGGGTAGA-3’ | | 134 |
|  | 30S ribosomal protein S11 | gi\|475576810 | | | AK063801 | F: 5’-TCCCGAAGAAGGATACCATGC-3’  R: 5’-TGAGAGCGCATGCAACATGA-3’ | | 125 |
|  | Malate dehydrogenase 1 | gi\|475608922 | | | AK101730 | F: 5’-AATGGCACCTGGAGTGACTG-3’  R: 5’-GTGAGGAACGCTGCTACCAT-3’ | | 103 |

**Table S1.** (*Continued*)

| Types of amplification | Names of protein species | Accession no. of the identified protein species | Accession no. of the genes encoding the identified protein species | Primer pairs | Amplified sizes (bp) |
| --- | --- | --- | --- | --- | --- |
| qPCR (rice) | Protein phosphatase 2C | gi|475573357 | Os04g33080 | F: 5’-TGTCAAACTACTGGGGCGTC-3’  R: 5’-CATGGTTTCCGTCAACTGCC-3’ | 114 |
|  | Lipoxygenase | gi|310656750 | AB099850 | F: 5’-AGCATCCCAGCACTAGAGGA-3’  R: 5’-TCCAAGCAACCTCGTTCTCC-3’ | 133 |
|  | Pyruvate orthophosphate dikinase | gi|32400838 | AK065739 | F: 5’-GTTCGGCAACGTCGTTATGG-3’  R: 5’-AATCCCTGGCAGTCAACTCG-3’ | 110 |
|  | Thioredoxin reductase | gi\|475477820 | Os03g0168500 | F: 5’-CTCGTTCATCTCAGCGTGGA-3’  R: 5’-TCTGCCAACCAGTTGAAGCA-3’ | 132 |
|  | Glutathione S-transferases | gi\|20067421 | AK064650 | F: 5’-ATACTCACACAGTTCGCGGC-3’  R: 5’-TACTCGTAGCTCAGGCCCTT-3’ | 143 |
|  | Late embryogenesis abundant protein | gi\|474359227 | U57641 | F: 5’-ACGCCGTGAATGATTTCCCT-3’  R: 5’-CACCCGTCAGAAATCCTCCC-3’ | 148 |
|  | Heat shock protein | gi\|11561808 | AK067414 | F: 5’-CACACCGTCCACCTGACTAC-3’  R: 5’-CTTCTTGGAGGGGTCCTTGG-3’ | 129 |
|  | 6-phosphofructokinase | gi\|474012166 | AK071798 | F: 5’-ATACTCACACAGTTCGCGGC-3’  R: 5’-TACTCGTAGCTCAGGCCCTT-3’ | 143 |
|  | Alcohol dehydrogenase | gi\|119388715 | AK061267 | F: 5’-AGAGTGTTGGAGAGTGTGCC-3’  R: 5’-CCCGTTGATGGAAAAGCGTG-3’ | 122 |
|  | Citrate synthase | gi\|475609802 | AF302906 | F: 5’-GCTGCCGACAGCAGTTAAAG-3’  R: 5’-TGACCTGGAACACTCGAACG-3’ | 138 |
|  | 20S proteasome beta 4 subunit | gi\|52548238 | AB032062 | F: 5’-CCTCTGCCAACTCGACTACG-3’  R: 5’-ACAGGTTCGTGATGGTCGTC-3’ | 143 |
|  | Aminotransferase | gi\|475548802 | AK060423 | F: 5’-TGATTGCCCGGCTTTACACT-3’  R: 5’-AACGCATTTGTGGTTGGCTC-3’ | 103 |

**Table S1.** (*Continued*)

| Types of amplification | Names of protein species | Accession no. of the identified protein species | Accession no. of the genes encoding the identified protein species | Primer pairs | Amplified sizes (bp) |
| --- | --- | --- | --- | --- | --- |
| qPCR (rice) | Cysteine synthase | gi|473980366 | AF073695 | F: 5’-CACGCGTCCGCAAGGA-3’  R: 5’-GCGTGTTCCCAATCAACTCG-3’ | 115 |
|  | 4-coumarate--CoA ligase-like protein | gi\|475508101 | AK120964 | F: 5’-ATCAATGTGCATGGCGTTCG-3’  R: 5’-CCCTGATGCAAATCTCCCCC-3’ | 149 |
|  | Annexin 4 | gi\|326489251 | AU031467 | F: 5’-GAGAAGCTGTCCGGGTTCAG-3’  R: 5’-GCCCACATCACCATCAGGTT-3’ | 124 |
|  | 6-phosphogluconolactonase | gi\|475537377 | AK069696 | F: 5’-CGCCTTGGATGGGTTTCTCT-3’  R: 5’-GCGACATTGCAATCACACCA-3’ | 146 |
|  | Adenylate kinase | gi\|475561713 | AK066688 | F: 5’-CTGGTGCACATTTCTGCTGG-3’  R: 5’-GGAACCAGCTGACCCTTCTC-3’ | 104 |
|  | CIPK-like protein | gi\|32442210 | AY256847 | F: 5’-ATGCGCATGGAGAACCTGAA-3’  R: 5’-ACTCCAGAGTGTCCCCCTTT-3’ | 124 |
|  | Translation initiation factor IF-3 | gi\|357137407 | AK069347 | F: 5’-TGGTGTGATGTGTGAGGTCG-3’  R: 5’-CATCGCGTAAAGCGAGCAAT-3’ | 118 |
| Internal control gene (rice) | 18S rRNA |  | NC_007886.1 | F: 5’-CTACGTCCCTGCCCTTTATACA-3’  R: 5’-ACACTTCACCGGACCATTCAA-3’ | 108 |
|  | OsUBQ5 |  | NC_029261.1 | F: 5’- ACCACTTCGACCGCCACTACT-3’  R: 5’- ACGCCTAAGCCTGCTGGTT-3’ | 112 |

Notes: F, forward primers; R, reverse primers.
