# Supplementary material for: Large-scale Proteomics Combined with Transgenic Experiments Demonstrates An Important Role of Jasmonic Acid in Potassium Deficiency Response in Wheat and Rice
Source: Mol Cell Proteomics. 2017 Aug 18;16(11):1889–905. doi: 10.1074/mcp.RA117.000032 (PMC5671998; doi:10.1074/mcp.RA117.000032)
Supplement: Supplemental Data [file supp_RA117.000032_4824_0_supp_3807_9tkkw3.doc]

**Method S1. 2D LC-MS/MS analysis and protein identification of iTRAQ proteomic experiment**

The peptides were separated by strong cation-exchange chromatography, desalted using a C18 Cartridge (Sigma, USA), and then subjected to further LC-MS/MS analysis. For MS analysis, we used the cHiPLC Nanoflex microchip system (Dublin, CA, USA) with nanoLC-MS/MS (Nano Ultra 2D Plus, Eksigent) equipped with an AB SCIEX TripleTOF 5600 MS (Toronto, Concord, Canada) (Kang et al., 2015). The MS/MS spectra were searched using MASCOT engine software (Matrix Science, London, UK; version 2.2) embedded in Proteome Discoverer 1.4 software (Thermo Electron, San Jose, CA). A database search was performed against the NCBInr database (http://blast.ncbi.nlm.nih.gov) (released June 10, 2015, 4,075,097 entries). In addition, the identified protein species were filtered using the cRAP database (ftp://ftp.thegpm.org/fasta/cRAP), with the following parameters: type = MS/MS ion search, enzyme = trypsin, mass values = monoisotopic, max missed cleavage = 2, fixed modification: carbamidomethyl (C), iTRAQ8plex (N-term), iTRAQ8plex (K), variable modification: oxidation (M), peptide mass tolerance = ± 20 ppm, fragment mass tolerance = 0.1 Da, peptide false discovery rate (FDR) ≤ 0.01 (1). The peptide for quantification was automatically selected by the ProGroup algorithm (AB SCIEX) to calculate the reporter peak area and showed in Data S1 and S2. In each experiment, bias correction for unequal mixing of the different labeled samples was performed. This correction was based on the assumption that the expression of most proteins does not have prominent change. Thus, if samples from each experimental condition were not combined in exactly equal amounts, bias correction fixed this systematic error. The software identified the median average protein ratio and corrected it to unity, and then applied this factor to all quantification results (2). Only protein species identified in all three biological replicates (Data S3) were considered for further analysis. To evaluate the repeatability of the replicates, we compared the protein abundance among three independent biological replicates and compared the ratios for each protein in each comparison with those of control (3). We then calculated and analyzed the ratios of the identified proteins overlapping in all three biological replicates. The abundance of a fold change ≥+1.20 or -1.20 regulated protein species using Duncan’s multiple range test (*P <* 0.05) was applied to the three biological replicates to identify significant differences, and multiple testing corrections were then used to adjust *P* values for controlling the false discovery rate. Furthermore, the ratios have been analyzed by Benjamini and Hochberg (BH) test (2-5). NCBInr and International Wheat Genome Sequencing Consortium (IWGSC) databases were used to search for the function/biological process, then the identified proteins were grouped them on the basis of their biological functions from Gene Ontology (GO) terms (Data S4). The mass spectrometry proteomics data have been deposited in the ProteomeXchange Consortium via the PRIDE partner repository (6).

**References:**

1. Sandberg, A., Lindell, G., Källström, B. N., Branca, R. M., Danielsson, K. G., Dahlberg, M., Larson, B., Forshed, J., and Lehtiö, J. (2012) Tumor proteomics by multivariate analysis on individual pathway data for characterization of vulvar cancer phenotypes. *Mol* *Cell Proteomics* 11, M112.016998.
2. Tomizioli, M., Lazar, C., Brugière, S., Burger, T., Salvi, D., Gatto, L., Moyet, L., Breckels, L., Hesse, A.M., Lilley, K.S., Seigneurin-Berny, D., Finazzi, G., Rolland, N., and Ferro, M. (2009) Deciphering thylakoid sub-compartments using a mass spectrometry-based approach. *Mol Cell Proteomics* 13, 2147–2167.
3. Cao, X., Fu, Z., Zhang, M., Han, Y., Han, H., Han, Q., Lu, K., Hong, Y., and Lin, J. (2016) iTRAQ-based comparative proteomic analysis of excretory–secretory proteins of schistosomula and adult worms of *Schistosoma japonicum*. *J Proteomics* 138, 30–39.
4. Chu, P., Yan, G. X., Yang, Q., Zhai, L. N., Zhang, C., Zhang. F, Q., and Guan, R. Z. (2015) iTRAQ-based quantitative proteomics analysis of *Brassica napus* leaves reveals pathways associated with chlorophyll deficiency. *J Proteomics* 113, 244–259.
5. Hu, X., Li, N., Wu, L., Li, C., Li, C., Zhang, L., Liu, T., Wang, W. (2015) Quantitative iTRAQ-based proteomic analysis of phosphoproteins and ABA-regulated phosphoproteins in maize leaves under osmotic stress. *Sci Rep* 5, 15626.
6. Ternent, T., Csordas, A., Oi, D., Gómez-Baena, G., Beynon, R.J., Jones, A.R., Hermjakob, H.H., and Vizcaino, J.A. (2014) How to submit MS proteomics data to proteomeXchange via the PRIDE database. *Proteomics* 14, 2233–2241.
