# Supplementary material for: Large-scale Proteomics Combined with Transgenic Experiments Demonstrates An Important Role of Jasmonic Acid in Potassium Deficiency Response in Wheat and Rice
Source: Mol Cell Proteomics. 2017 Aug 18;16(11):1889–905. doi: 10.1074/mcp.RA117.000032 (PMC5671998; doi:10.1074/mcp.RA117.000032)
Supplement: Supplemental Data [file supp_RA117.000032_4824_0_supp_3808_ftkkw3.doc]

**Method S2.** qPCR analysis

Total RNA from root and leaf tissues of wheat seedlings or the rice transgenic lines was extracted using TRIzol reagent (Invitrogen, Carlsbad, CA, USA), and then treated with RNase-free DNase I (Takara Biotechnology [Dalian] Co., Ltd., Dalian, China) to remove contaminating genomic DNA. First-strand cDNAs were synthesized from 2 μg of total RNA, oligo(dT)_18_ primer, and 100 units of Super-Script II reverse transcriptase (Invitrogen, Carlsbad, CA, USA). The amplified cDNA sequences were isolated, and purified using Geneclean Kit (Takara Biotechnology [Dalian] Co., Ltd., Dalian, China), and cloned into pMD20-T vector (Takara Biotechnology [Dalian] Co., Ltd., Dalian, China). Each product was completely sequenced using the Applied Biosystems 3,710 DNA capillary sequence three times. qPCR reactions contained 10 µL of FastStart Universal SYBR Green Master, 1 μL of cDNAs dilution, 250 nM each primer, and distilled water up to 20 µL. Cycle parameters were 95°C for 10 min, 40 cycles of 95°C 15 s, 55°C to 58°C for 60 s. Wheat *actin* and *phosphoglyceraldehyde dehydrogenase* (*GAPDH*) (GenBank accession no. AB181991 and EU022331) were used as two internal control genes to measure the transcription levels of the genes encoding 12 wheat K^+^-responsive protein species (1). Moreover, rice *18S rRNA* and ubiquitin (GenBank accession no. NC_007886 and NC_029261) genes (2), another two internal control genes, were used to evaluate the transcription levels of the selected genes in both rice WT and transgenic lines expressing *TaAOS* gene. Names of the selected genes and their primers are listed in Table S1. Relative transcript levels were calculated using the 2-^∆∆Ct^ method (3).

**References:**

1. Wei, L., Wang, L., Yang, Y., Liu, G., Wu, Y., Guo, T., and Kang, G. (2015) Abscisic acid increases leaf starch content of polyethylene glycol-treated wheat seedlings by temporally increasing transcripts of genes encoding starch synthesis enzymes. *Acta Physiol Plant* 37, 206.
2. Wang, R., Jing, W., Xiao, L., Jin, Y., Shen, L., Zhang, W. (2015) The rice high-affinity potassium transporter1;1 is involved in salt tolerance and regulated by an MYB-type transcription factor. *Plant Physiol* 168, 1076–1090.
3. Schmittgen, T.D., and Livak, K.J. (2008) Analyzing real-time PCR data by the comparative C(T) method. *Nature Protocols* 3, 1101–1108.
